# Ammoxidation of Unprotected Glycosides: A One‐Pot Conversion of Alcohols to Nitriles

**DOI:** 10.1002/chem.202500796

**Published:** 2025-04-21

**Authors:** Jacob‐Jan Haaksma, J. Prathap Kaniraj, Wiktoria M. Opielak, June van Egmond, C. Maurits de Roo, Wesley R. Browne, Adriaan J. Minnaard, Martin D. Witte

**Affiliations:** ^1^ Stratingh Institute for Chemistry University of Groningen 9747 AG Groningen The Netherlands

**Keywords:** ammoxidation, nitrile sugars, site‐selective modification, unprotected carbohydrates

## Abstract

Functionalized carbohydrates are important in various fields, but protection‐free selective functionalization often remains challenging. We demonstrate that the primary hydroxy group in minimally protected carbohydrates can be directly converted into a nitrile group with TEMPO, PIDA, and ammonium acetate. Both nitrile hexopyranoses and nitrile pentofuranoses are obtained and subsequent derivatizations of the nitrile group to other versatile functional groups are demonstrated. Combined evidence from literature and in‐situ reaction progress monitoring with Raman spectroscopy led to the proposal that iminoiodinanes derived from PIDA play an important role in the mechanism of ammoxidation.

## Graphical abstract

Efficient C5‐selective ammoxidation of minimally protected carbohydrates enables direct access to nitrile sugars, valuable in bioorthogonal chemistry and drug development. Using TEMPO, PIDA, and ammonium acetate in aqueous acetonitrile, this method offers high selectivity without protecting groups. Mechanistic insights reveal iminoiodinane intermediates, explaining the reaction's remarkable precision.

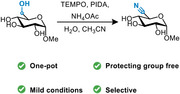

## 1 Introduction

Functionalized carbohydrates are used in a wide range of applications. For example, carbohydrates equipped with bioorthogonal handles are employed in metabolic labeling studies.^[^ ^1^, ^2^, ^3^, ^4^ ^]^ Several well‐known drugs, among which are important antidiabetics, primarily consist of functionalized carbohydrates.^[^ ^5^, ^6^, ^7^ ^]^ In addition, modified oligo‐ and polysaccharides are valuable precursors for bio‐based materials.^[^ ^8^, ^9^, ^10^ ^]^ Despite their value, the preparation of functionalized carbohydrates remains a hurdle. Differentiation between the multiple hydroxyl groups is often difficult due to their similar reactivities and the hydrophilic nature of unprotected carbohydrates reduces their solubility in organic solvents. As a result, conventional modification techniques mostly rely on protecting group strategies.^[^ ^11^ ^]^ Consequently, even seemingly straightforward modifications require multiple synthetic steps. These can be circumvented by site‐selective functionalization methods, improving both step economy and atom economy.

Over the past years, various methods to functionalize minimally protected carbohydrates have been developed,^[^ ^12^ ^]^ with various contributions from our group.^[^ ^13^, ^14^, ^15^ ^]^ Interestingly, despite the great versatility of the nitrile group as a synthetic and bioorthogonal handle,^[^ ^16^, ^17^, ^18^, ^19^ ^]^ methods to site‐selectively equip hexopyranosides and pentofuranosides with a C5‐cyano group are currently lacking. Such a C5‐nitrile may find use both as a click handle^[^ ^18^ ^]^ and as a Raman spectroscopic tag,^[^ ^20^ ^]^ while also paving the way for the development of glucuronide bioisosteres^[^ ^21^ ^]^ and novel glycosidase inhibitors.^[^ ^22^, ^23^ ^]^ Moreover, carbohydrates with aldehydes, amines, carboxylic acids, and heterocycles at the C5‐position can be quickly accessed from these unprotected sugar nitriles.

**Scheme 1:**
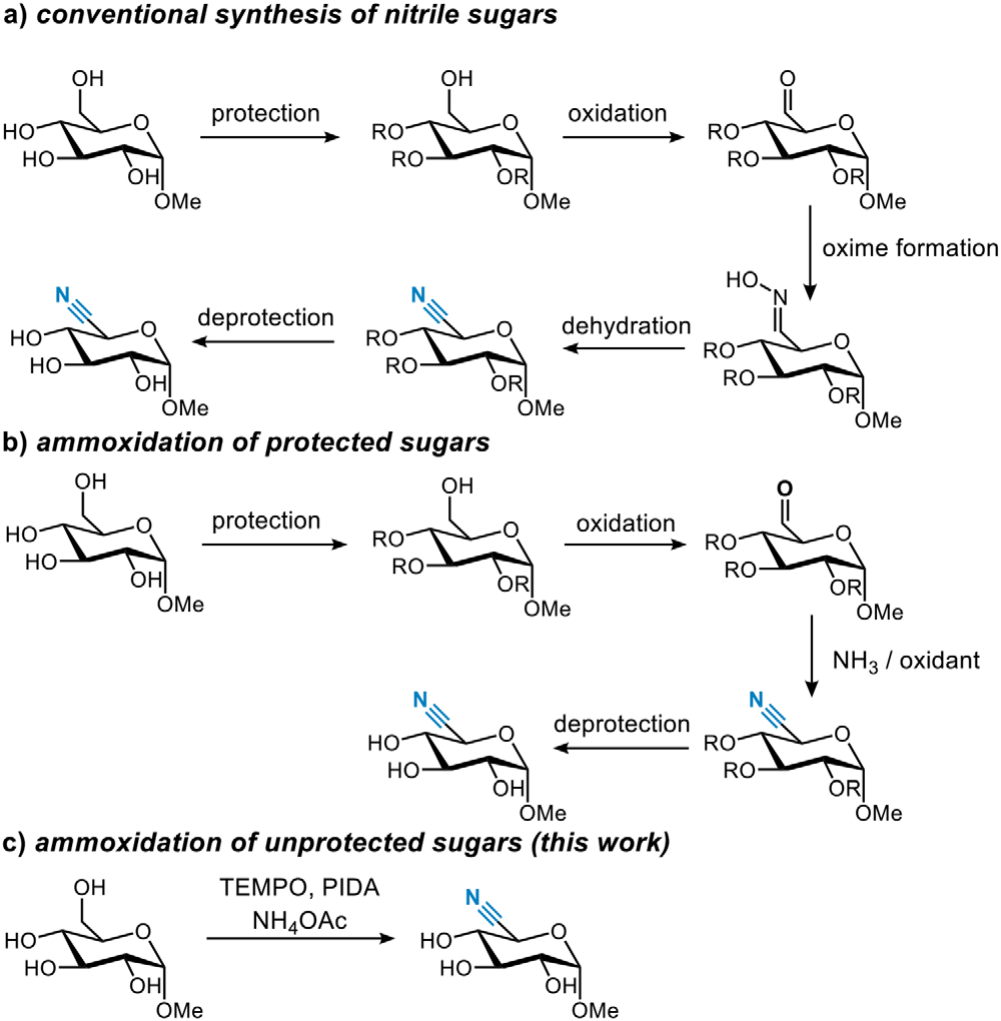
Overview of previous and current work. (a) Conventional synthesis of nitrile sugars via oxime formation and dehydration; (b) ammoxidation of protected sugars; (c) our proposed strategy for the ammoxidation of unprotected sugars.

Typically, C5‐sugar nitriles are synthesized by the protection of the secondary hydroxy groups, oxidation of the primary alcohol at C6 to the corresponding aldehyde, aldoxime formation, dehydration to the nitrile, and finally deprotection (Scheme 1a).^[^ ^24^ ^]^ However, such dehydrating conditions are incompatible with unprotected carbohydrates. A less well‐explored approach to synthesizing sugar nitriles is ammoxidation (Scheme 1b).^[^ ^23^, ^25^, ^26^ ^]^ In this approach, the alcohol or aldehyde at the C6‐position is converted into the nitrile using a combination of ammonia and an oxidant in a single step.

Thus far, only a handful of C5‐nitrile pyranosides have been prepared using ammoxidation and, in all examples but one, all secondary hydroxy groups were protected.^[^ ^25^ ^]^ Protecting group strategies should not be necessary, provided that (1) the C6‐OH can be oxidized selectively in the presence of the secondary hydroxy groups and (2) the competing side reaction in water, in which the aldehyde intermediate is oxidized to a carboxylic acid,^[^ ^27^ ^]^ can be suppressed. The validity of this reasoning is illustrated by the work of Vatèle,^[^ ^25^ ^]^ who exploited the high selectivity of (2,2,6,6‐tetramethylpiperidin‐1‐yl)oxidanyl (TEMPO)/co‐oxidant systems for primary alcohols to establish an ammoxidation method that directly converts primary alcohols into nitriles. The reported TEMPO/phenyliodine(III) diacetate (PIDA)/NH_4_OAc system was successfully applied to a partly protected methyl‐α‐D‐glucopyranoside with a free primary and a secondary hydroxy group. The ammoxidation reaction was performed in a mixture of acetonitrile (ACN)/water (9:1). Under these conditions, competing glucuronate formation was minimal.

Inspired by the work of Vatèle,^[^ ^25^ ^]^ we aimed to extend this ammoxidation approach to minimally protected carbohydrates (Scheme 1c). This required a re‐investigation of the solvent system, balancing between the solubility of the carbohydrates and the ammonium acetate on one hand, and minimizing acid formation on the other hand. Moreover, this reaction should be combined with a suitable isolation procedure as the products are water‐soluble.

## 2 Results and Discussion

The addition of at least 50% water was required to reach a reasonable concentration of methyl α‐D‐glucopyranoside **1** in aqueous acetonitrile (Table 1). Gratifyingly, we observed the formation of C5‐nitrile **2** upon reaction with TEMPO, PIDA, and 4 equiv. of ammonium acetate. However, a considerable amount of methyl α‐D‐glucuronate **3** was observed as well, indicating that, indeed, oxidation of the aldehyde hydrate was competing with the ammoxidation reaction. In addition, 5% of a side product identified as the C5‐epimer of **2** was observed (see Chapter 7 in Supporting Information).

**Table 1:** Reaction optimization of the ammoxidation of **1**.

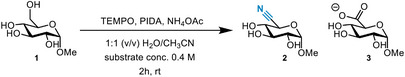					
Entry	TEMPO (mol%)	NH_4_OAc (eq.)	PIDA (eq.)	Conversion^[^ ^a^ ^]^	2:3^[^ ^a^ ^]^
1	5	10	3	38%	99:1
2	10	10	3	100%	20:1
3	10	5	3	100%	10:1
4	10	1.5	3	85%	5:1
6	10	15	3	98%	50:1
**7**	**10**	**10**	**2.5**	**100%**	**20:1**
8	10	10	2.1	90%	20:1

^[a]^ Determined by ^1^H‐NMR (CD_3_OD).

While the reaction proceeds in pure water, the selectivity is difficult to control as excessive amounts of ammonium acetate were required to suppress acid formation to a reasonable level. Hence, we continued with a 1:1 ACN/water mixture. We performed optimization studies with **1** to increase the selectivity of the reaction, a selection of the results is highlighted in Table 1. Decreasing the TEMPO loading below 10 mol% led to a drastic decrease in conversion. Moreover, the concentration of ammonium acetate appeared to be strongly correlated with the selectivity of this reaction, as a higher ammonium acetate concentration resulted in a more selective formation of nitrile **2** over glucuronate **3**. At 10 equiv. of ammonium acetate, the reaction proceeded satisfactorily, even though phase separation of the aqueous and organic phases was observed. Saturating the system with 15 equiv. of ammonium acetate resulted in a further increase in selectivity but at the cost of a lower conversion. The PIDA loading could be lowered from 3 to 2.5 equiv. Based on these data, we proceeded with the reaction conditions as outlined in Entry 7.

**Figure 1:**
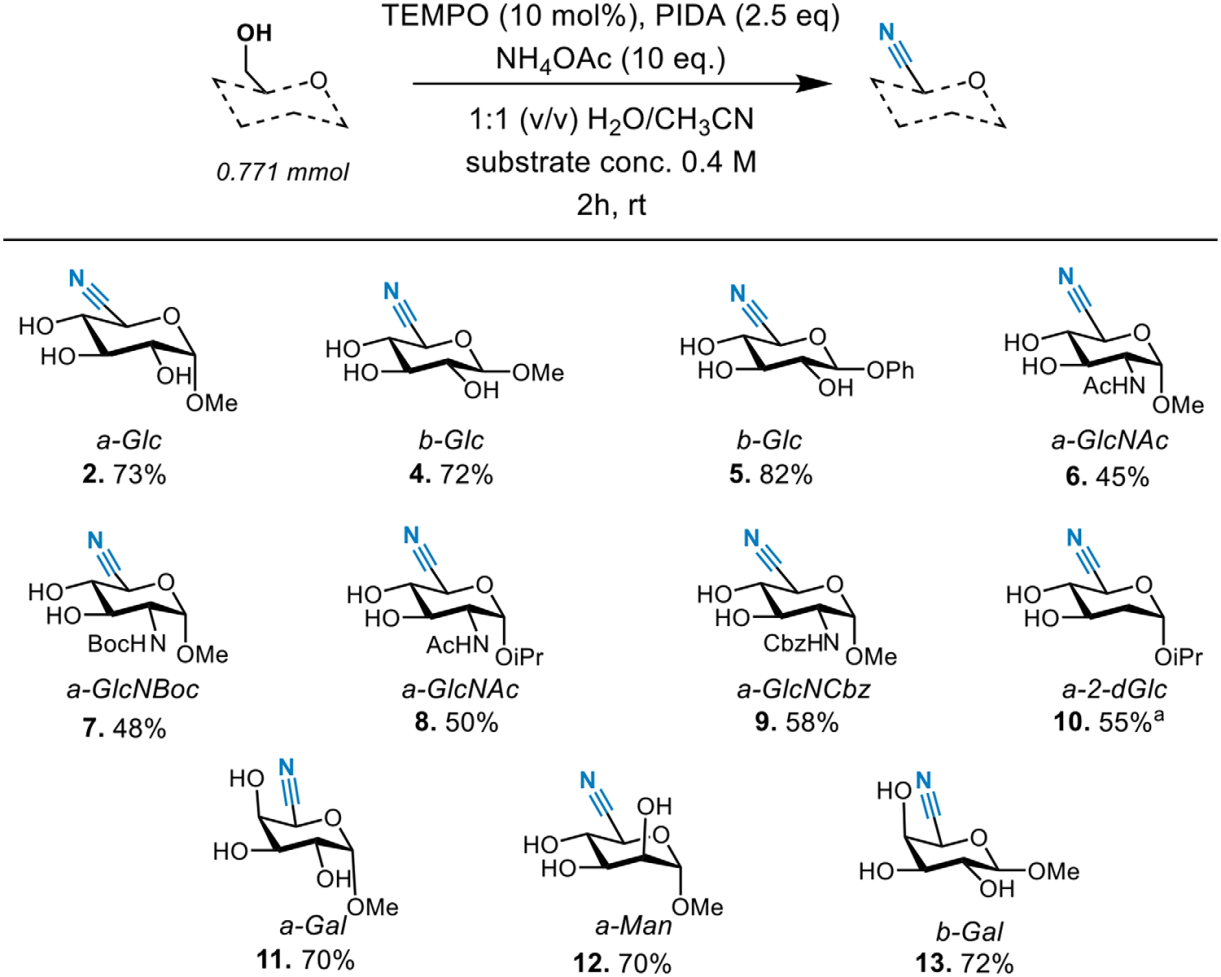
Ammoxidation of various unprotected hexopyranosides. Percentages expressed as isolated yield. Isolated as a mixture of epimers.

Having established the optimized reaction conditions, we sought to expand our protocol to other unprotected sugars. In addition to α‐glucopyranosides, it was found that β‐glucopyranosides (**4**, **5**) also performed well (Figure 1). In addition, 2‐deoxy‐2‐amino sugars (**6**, **7**, **8**, **9**) and 2‐deoxy sugars (**10**) were efficiently converted into the corresponding C5‐nitrile sugars with this protocol. While galactopyranosides and mannopyranosides (**11**, **12**, **13**), unlike the other substrates, did not reach full conversion after 2 h, the respective nitrile sugars were isolated in good yields.

Pentofuranosides did not yield nitrile‐containing products. It is known in the literature that unprotected pentofuranosides—in contrast to their protected counterparts—are often challenging substrates for TEMPO/PIDA‐mediated oxidation, as numerous over‐oxidation products can be formed.^[^ ^28^ ^]^ The stereochemistry of the hydroxy group at the C2 and C3 positions of unprotected furanosides significantly influences the reaction outcome of the PIDA/TEMPO oxidation. A cis configuration (e.g., ribofuranoses) provides no reaction or decomposition. Substrates with a trans configuration (e.g., arabinofuranoses) and 2‐deoxy furanosides react successfully. As such, the only furanosides that have been successfully oxidized to the corresponding carboxylic acid using TEMPO are O‐methyl arabinofuranoside, 2‐deoxy uridine, and arabino uridine.^[^ ^27^ ^]^ Surprisingly, however, these substrates did not react in the TEMPO‐mediated ammoxidation.

**Scheme 2:**
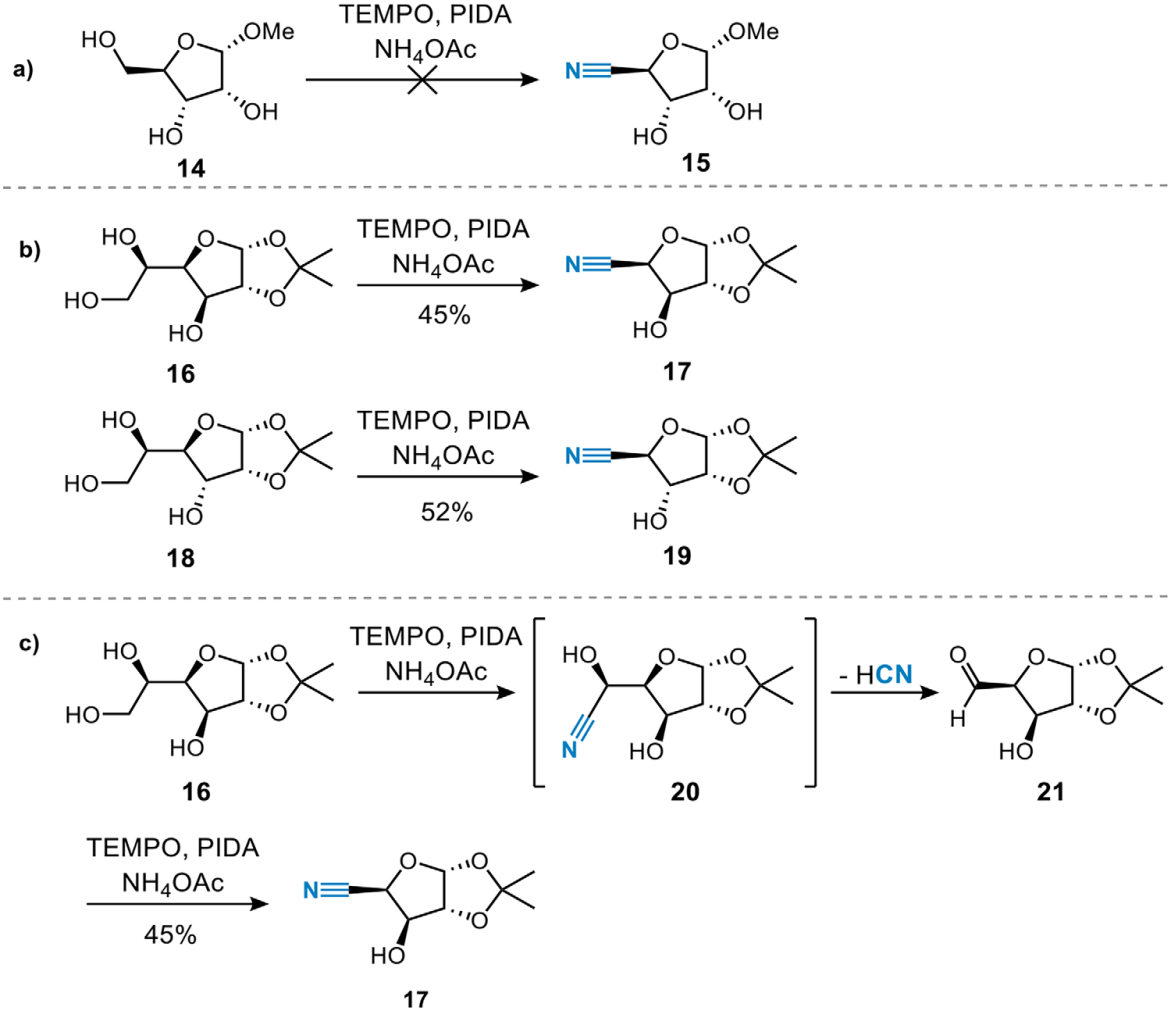
(a) Pentofuranosides are not ammoxidized (b) Ammoxidation of hexofuranosides leads to the pentofuranoside nitrile; (c) Proposed mechanism of the ammoxidation of hexofuranosides. Percentages are isolated yields.

Since pentofuranosides such as ribose **14** did not react to form the nitrile product (Scheme 2a), we shifted our attention to hexofuranosides. When we performed the ammoxidation reaction on hexofuranoside **16**, we observed a high conversion (Scheme 2b), but interestingly not the hexofuranoside nitrile was obtained, but rather the 4‐cyano xylofuranoside **17**. A significant loss in yield was caused by the removal of the isopropylidene‐protecting group during the course of the reaction. We hypothesize that the pentoside is formed via a mechanism similar to the classic Wohl degradation.^[^ ^29^ ^]^ Ammoxidation of glucofuranose yields a cyanohydrin (Scheme 2c). Elimination of the cyano‐hydrin affords the respective aldehyde which undergoes a second ammoxidation resulting in 4‐cyanofuranoside **17**. Three equivalents of co‐oxidant are consumed in this sequence and indeed the yield improved when we increased the PIDA loading. Similar results were obtained when the reaction was performed on allofuranoside **18**. This approach provides a versatile alternative to access these pentafuranoside nitriles, which could prove highly valuable for the preparation of modified furanosides. These compounds are frequently utilized in chemical biology and serve as essential building blocks for the development of antiviral agents.^[^ ^30^, ^31^ ^]^

With a versatile protocol established, we demonstrated its applicability by scaling up the reaction. The reaction worked equally well on a 1.5‐g scale. As purification of minimally protected carbohydrates by silica gel column chromatography is often low‐yielding, we sought a way to circumvent this. Purification of the product would require the separation of excess PIDA and ammonium acetate, as well as phenyl iodide and TEMPO.

We serendipitously found that unreacted PIDA precipitated after the addition of isopropanol to the concentrated crude reaction mixture. This PIDA was readily removed by filtration and could be used in a later reaction. To remove the formed phenyl iodide, the filtrate was then concentrated in vacuo and triturated with toluene. Lastly, the residual ammonium acetate was removed by repeated co‐evaporation with *n*‐butanol to afford 5‐cyano glucopyranoside in 97% yield with trace amounts of *n*‐butanol and the C5 epimer.

**Scheme 3:**
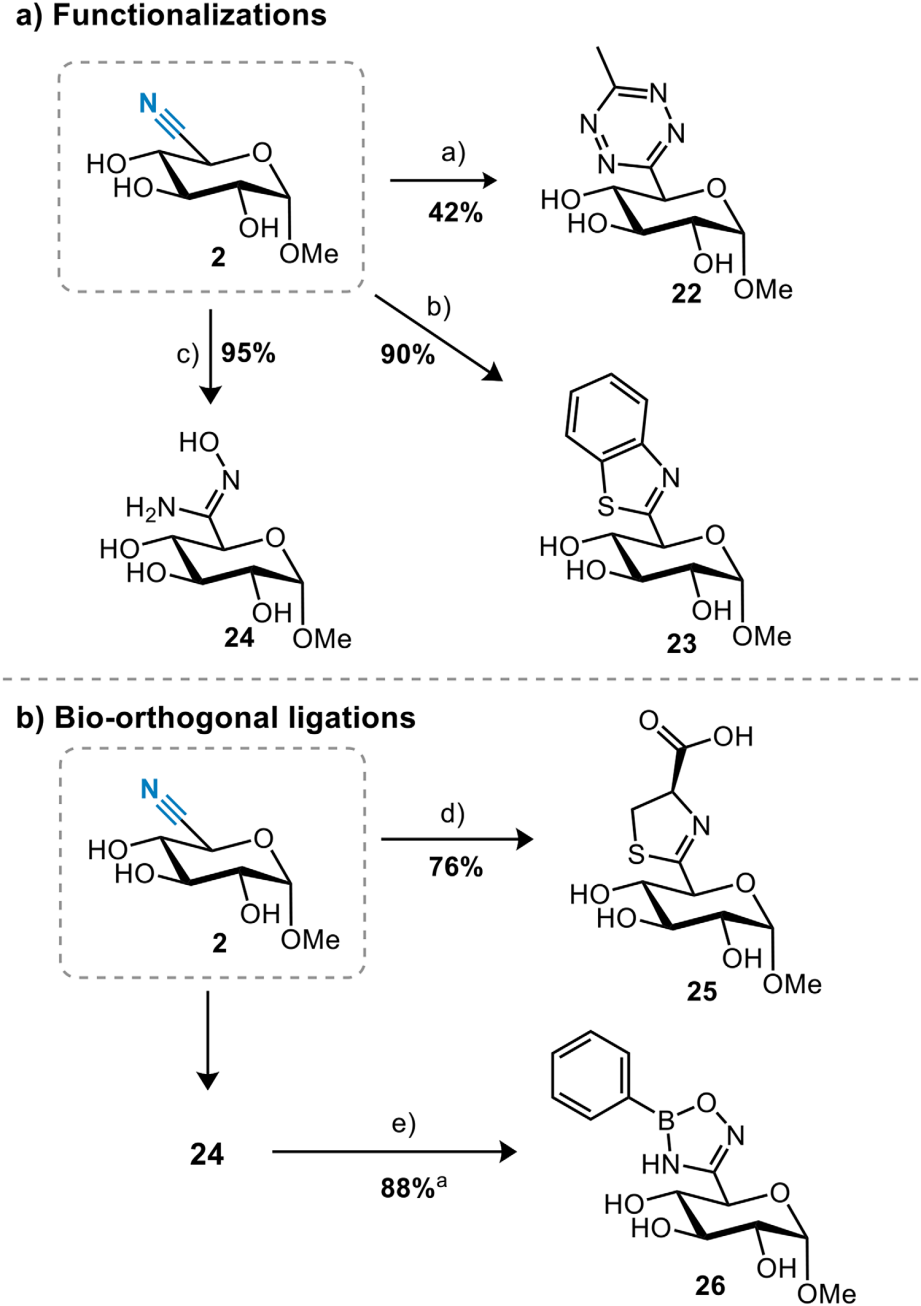
Several derivatizations of nitrile sugar 2. (a) CH_3_CN, 3‐mercaptopropionic acid, N_2_H_4_ • H_2_O, then NaNO_2,_ HCl; (b) 2‐aminothiophenol, KHCO_3_; (c) NH_2_OH; (d) L‐cysteine, NaHCO_3_; (e) PhB(OH)_2._, DMSO‐d_6_. Percentages are isolated yields. ^a^Conversion determined by crude ^1^H‐NMR (DMSO‐d_6_). ^a^Percentage expressed as crude conversion as determined by ^1^H‐NMR.

To demonstrate the versatility of the nitrile group in **2**, we performed various derivatization reactions.^[^ ^19^, ^32^, ^33^, ^34^ ^]^ From nitrile **2**, tetrazine **22**, benzothiazole **23**, and amidoxime **24** were readily accessed (Scheme 3). These derivatives may play a role in future bioorthogonal reactions, especially tetrazines have gained considerable attention in the context of the labeling and delivery of probes and drugs.^[^ ^34^ ^]^ The possibility for bioorthogonal ligations was also demonstrated by a click‐reaction with L‐cysteine^[^ ^35^ ^]^ to form **25** and the in‐situ formation of the labile adduct with phenylboronic acid^[^ ^33^ ^]^ resulting in oxadiazoborole **27**. All these reactions were compatible with the unprotected secondary hydroxy groups.

The classical mechanistic sequence to describe this ammoxidation would follow a pathway of oxidation of the primary alcohol to the aldehyde, followed by imine formation and subsequent oxidation of the imine to afford the nitrile. However, this mechanism fails to explain the excellent selectivity of this reaction towards the nitrile product. Typically, the hydrate is formed after the TEMPO‐mediated oxidation of the primary alcohol to the aldehyde, leading to the subsequent oxidation of this hydrate to the carboxylic acid. However, despite the abundance of water, this is not the case in the current ammoxidation reaction. In addition, the presence of the side product identified as the C5‐epimerized nitrile can not be explained with this mechanistic sequence. These observations led us to believe that an alternative mechanism was at play and we were intrigued to investigate this further.

**Scheme 4:**
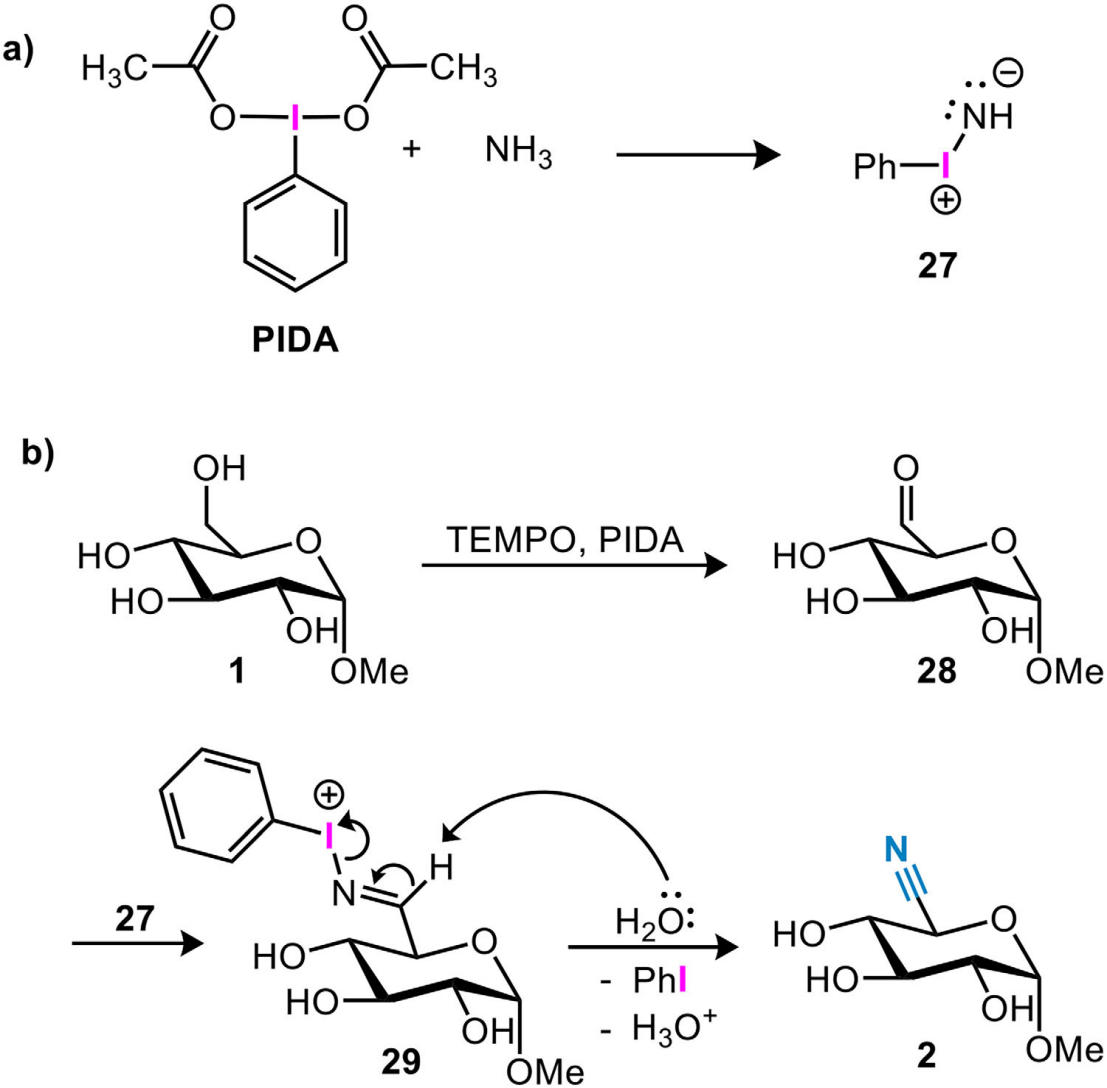
(a) The formation of iminoiodinane **27** from PIDA and ammonia. (b) Proposed mechanism of the ammoxidation reaction.

Literature suggests that PIDA and ammonium acetate react to form iminoiodinane **28** (Scheme 4a).^[^ ^36^ ^]^ The iminoiodinanes are best described as ylids and are somewhat reminiscent of the iminophosphoranes encountered in aza‐Wittig reactions. As such, we also expect iminoiodinanes to act as excellent nucleophiles. If such an iminoiodinane were to be present in the reaction mixture, imine formation of **27** with the sugar aldehyde would afford a phenyliodonium imine. With the reduction of the meta‐stable I(III) to a more stable I(I) species as an additional driving force, this intermediate is set up for a rapid elimination to afford the nitrile, explaining the selectivity of the ammoxidation.

**Figure 2:**
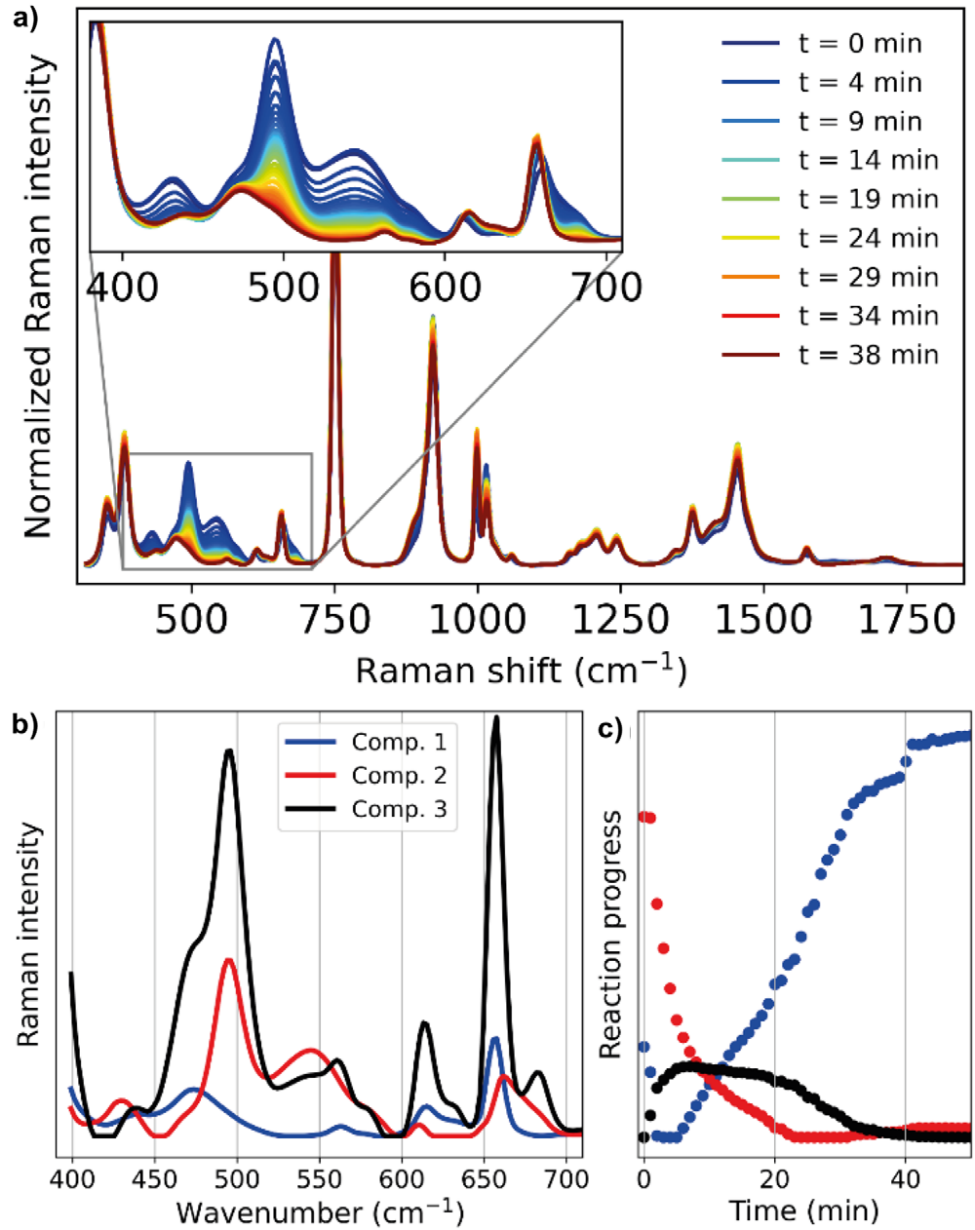
MCR analysis of the ammoxidation of **1** (a) Raman spectra over time. (b) The Raman traces in the mid‐wavenumber range of the components resulting from MCR analysis. (c) The relative contribution of these components over time.

In an effort to observe this species, the ammoxidation reaction was followed over time by in‐situ Raman spectroscopy (Figure 2a). To obtain reliable spectrometric results, the solvent system was changed to t‐BuOH/H_2_O/ACN (1:1:1) to prevent phase separation. A test reaction revealed that this change in the solvent system left the reaction outcome unchanged. The Raman spectra were subjected to multivariate curve resolution (MCR) to determine the minimum number of components to explain the data. The data were found to be explained well by 3 components corresponding well to the starting material, the final product, and a main intermediate species (Figure 2a,c). Hence, the red trace represents well the consumption of starting material **1**, the blue trace the formation of product **2**, and the black trace an intermediate species. We observed that the concentration of the intermediate component increases at the start of the reaction after which it reaches a plateau and decreases again. Although this species could correspond to the aldehyde intermediate, the absence of the characteristic aldehyde stretch around 1700 cm^−1^ suggests otherwise. Moreover, we do not expect this to be the respective hydrate as this would quickly be oxidized to the carboxylic acid, which we do not observe in significant amounts. Furthermore, MCR analysis in the mid‐wavenumber range does not reveal this species (only in the low‐wavenumber range), consistent with the expected high intensity of iodo‐related Raman bands. We therefore propose that the intermediate species is iminoiodinane **27** or the phenyl iodonium imine **29** derived thereof (Scheme 4). Considering this evidence, we propose a mechanism in which iminoiodinane **27** plays an important role (Scheme 4b). After the TEMPO‐mediated oxidation of the C6‐position of **1**, the respective aldehydo‐glucose **28** is obtained. Imine formation with iminoiodinane **27** then forms the phenyliodonium imine intermediate **29**. Deprotonation of the imine α‐proton then leads to the elimination of phenyl iodide and completes the ammoxidation affording **2**. The formation of the C5‐epimerized side product can now be explained as well. The generation of the phenyliodonium imine renders the proton at the C5 position significantly more acidic. Removal of this proton produces the corresponding enamine and re‐establishes the nitrogen‐iodine ylide. As the reverse reaction lacks stereospecificity, it accounts for the observed epimerization.

## 3 Conclusion

In conclusion, efficient and selective ammoxidation of minimally protected carbohydrates can be achieved using TEMPO, PIDA, and ammonium acetate in a 1:1 water/ACN solvent system. When applied to minimally protected hexopyranosides, the respective C5 nitrile sugars are obtained in good yields. Although pentofuranoses are unreactive under the reported conditions, the desired C5‐nitrile pentofuranosides can still be accessed by using hexofuranosides as substrates. Chain shortening of the initially formed cyanohydrin, followed by ammoxidation of the resulting aldehyde leads to the desired products. A 1.5‐g scale ammoxidation of methyl α‐D‐glucopyranoside combined with chromatography‐less purification shows the scalability of this reaction. Evidence from literature on the formation of iminoiodinanes from PIDA and ammonium acetate prompted us to investigate the possible role of this species in this reaction. In‐situ Raman spectroscopy revealed an intermediate species that we ascribed to the iminoiodinane or a derivative thereof. Incorporating iminoiodinanes into the mechanistic framework provides an explanation for both the formation of the C5‐epimerized nitrile side‐product and the remarkable selectivity of this reaction towards the nitrile, leading us to propose a mechanism where the iminoiodinane plays a pivotal role.

## Conflict of Interests

The authors declare no conflict of interest.

## Supporting information



Supporting Information

## Data Availability

The data that support the findings of this study are available in the supplementary material of this article.
