# Supplementary material for: Ammoxidation of Unprotected Glycosides: A One‐Pot Conversion of Alcohols to Nitriles
Source: Chemistry. 2025 Apr 21;31(28):e202500796. doi: 10.1002/chem.202500796 (PMC12089893; doi:10.1002/chem.202500796)
Supplement: Supplementary file 1 — Supporting Information [file CHEM-31-e202500796-s001.docx]

**Supporting Information**

**Ammoxidation of Unprotected Carbohydrates: A One-Pot Conversion of Alcohols to Nitriles**

Jacob-Jan Haaksma, J. Prathap Kaniraj*, Wiktoria M. Opielak, June van Egmond, C. Maurits de Roo, Wesley R. Browne, Adriaan J. Minnaard*, Martin D. Witte*

**Table of Contents**

[1. General Information S2](#_Toc194400910)

[2. General procedure for the ammoxidation S2](#_Toc194400911)

[3. Procedure for the large-scale ammoxidation of 1 S2](#_Toc194400912)

[4. Reaction optimization S3](#_Toc194400913)

[4.1 TEMPO loading optimization S3](#_Toc194400914)

[4.2 Ammonium acetate loading optimization S3](#_Toc194400915)

[4.3 PIDA loading optimization S4](#_Toc194400916)

[4.4 Ammonium source optimization S4](#_Toc194400917)

[5. Synthesis of Sugar Nitriles: Experimental Procedures and Characterization S5](#_Toc194400918)

[6. Synthesis of Sugar Nitrile Derivatives: Experimental Procedures and Characterization S9](#_Toc194400919)

[7. Identification of C5-epimer S12](#_Toc194400920)

[8. MCR analysis of Raman spectral data S14](#_Toc194400921)

[8.1 MCR analysis of Raman spectral data between 400-700 cm^-1^ S14](#_Toc194400922)

[8.2 MCR analysis of the spectral data between 975-1900 cm^-1^ S17](#_Toc194400923)

[9. ^1^H-NMR Spectra S21](#_Toc194400924)

[9.1 Ammoxidation S21](#_Toc194400925)

[9.2 Derivatizations S28](#_Toc194400926)

[9.2.1 Functionalizations S28](#_Toc194400927)

[9.2.2 Bioorthogonal Ligations S29](#_Toc194400928)

[10. ^13^C-NMR Spectra S31](#_Toc194400929)

[10.1 Ammoxidation S31](#_Toc194400930)

[10.2 Derivatizations S38](#_Toc194400931)

[10.2.1 Functionalizations S38](#_Toc194400932)

[10.2.2. Bioorthogonal Ligations S39](#_Toc194400933)

# 1. General Information

All solvents were purchased form commercially available sources and used without further purification. All chemicals were purchased from Sigma-Aldrich and used without further purification.

Flash column chromatography was performed manually with silica (SiliaFlash P60, 230-400 mesh, Silicycle). TLC was performed on Merck silica gel 60, 0.25 mm plates and visualization was done by staining with anisaldehyde stain (a mixture of AcOH (300 mL), H_2_SO_4_ (6 mL) and anisaldehyde (3 mL)).

NMR spectra (^1^H and ^13^C) were obtained with an Agilent MR400 spectrometer with reference to solvent residue peaks, i.e., for CDCl_3_ at 7.26 ppm for ^1^H and 77.16 ppm for ^13^C, for DMSO-d_6_ at 2.50 ppm for ^1^H and 39.52 ppm for ^13^C, for CD_3_OD at 3.31 ppm for ^1^H and 49.00 ppm for ^13^C and for D_2_O at 4.79 ppm for ^1^H. All data is reported as follows: Chemical shifts (δ), multiplicity (s = singlet, d = doublet, dd = doublet of doublets, t = triplet, q = quartet, m = multiplet) coupling constant J (Hz), and integration. High resolution mass spectra (HRMS) were recorded on a Thermo Scientific LTQ Orbitrap XL. Optical rotations were measured on a Schmidt+Haensch polarimeter (Polartronic MH8) with a 10 cm cell (c given in g/100 mL) at ambient temperature (±20 °C). Melting points were recorded using a Buchi Melting Point M-560.

# 2. General procedure for the ammoxidation

To a pressure tube equipped with a stirring bar was added PIDA (1.21 g, 1.93 mmol, 2.5 eq.), TEMPO (24 mg, 0.08 mmol, 10 mol%), ammonium acetate (1.16 g, 7.71 mmol, 10 eq.) and minimally protected carbohydrate (0.77 mmol, 1 eq.). Acetonitrile (1 mL) and water (1 mL) were added to the mixture resulting in a biphasic system. The mixture was allowed to stir for 2 h at room temperature. The crude reaction mixture was analyzed by ^1^H-NMR. Purification was done by step gradient flash column chromatography. The eluent consisted of a mixture of methanol in DCM ranging from 5 to 15% methanol, depending on the substrate. Pure product was characterized by ^1^H-NMR, ^13^C-NMR and HRMS.

# 3. Procedure for the large-scale ammoxidation of 1

To a flask equipped with a stirring bar was added PIDA (6.22 g, 19.3 mmol, 2.5 eq.), TEMPO (120 mg, 0.77 mmol, 10 mol%), ammonium acetate (5.95 g, 77.2 mmol, 10 eq.) and methyl α-D-glucopyranoside **1** (1.50 g, 7.72 mmol, 1 eq.). Water (10 mL) and acetonitrile (10 mL) were added to the mixture, which resulted in a biphasic system. The reaction had reached approximately 80% conversion after 2.5 h and was left to stir overnight at room temperature to reach full conversion after which the mixture was concentrated *in vacuo*. The concentrate was dissolved in isopropanol and the resulting precipitate was filtered off. The filtrate was concentrated *in vacuo*. The remaining oil was triturated with toluene (3 x 10 mL) and co-evaporated with *n*-butanol (5 x 20 mL), after which evaporation of all volatiles resulted in the product with 24 mol% of *n*-butanol remaining. Product **2** was obtained in 97% yield (1.56 g, 7.50 mmol) and appeared as a brown amorphous solid. The product was characterized by ^1^H-NMR and ^13^C-NMR.

# 4. Reaction optimization

## 4.1 TEMPO loading optimization

Lowering the TEMPO loading below the initial 10 mol% was found to have a negative impact on the conversion of the reaction.

***Table S1.*** *Optimization of the TEMPO loading.*

**
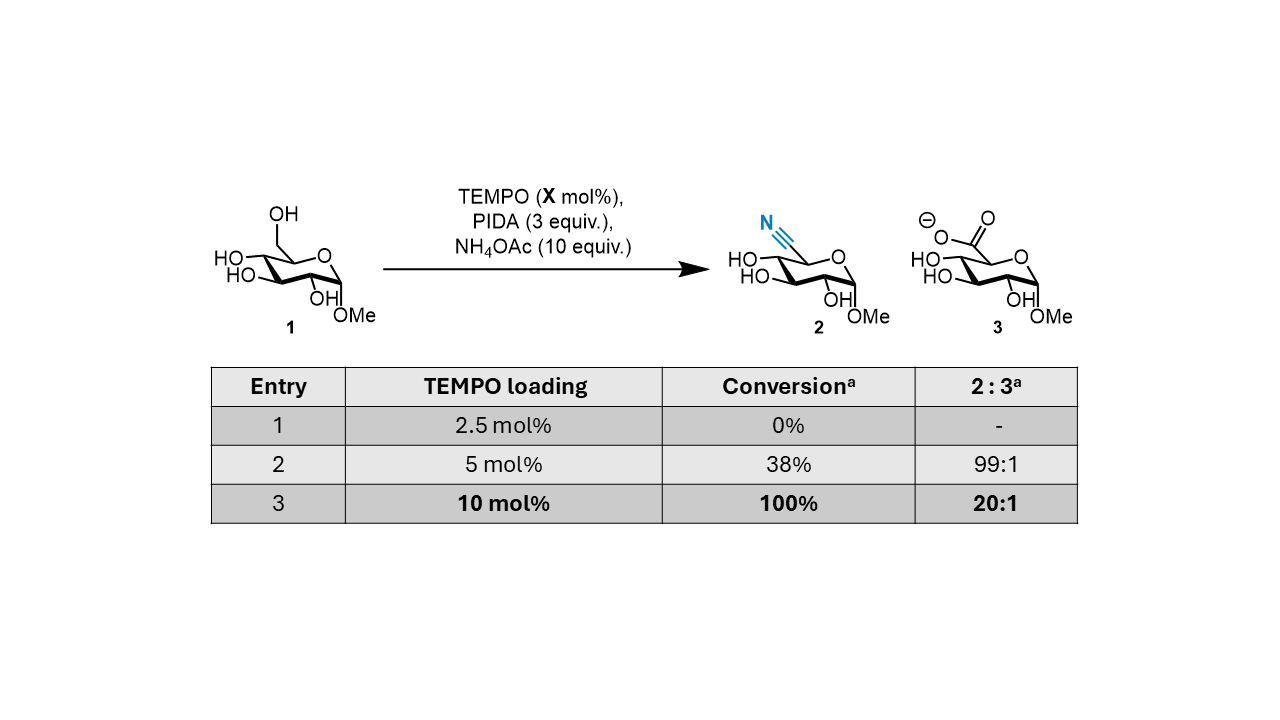
**

*Reactions were performed on 0.77 mmol scale, in 2 mL 1:1 (v/v) CH_3_CN/H_2_O. Stirring for 2 h at room temperature. ^a^As determined by crude ^1^H-NMR (400 MHz, CD_3_OD).*

## 4.2 Ammonium acetate loading optimization

Decreasing the ammonium acetate concentration below 10 eq. shifts the product distribution more to the glucuronate side-product. Ammonium acetate loadings exceeding 10 eq. was observed to be detrimental to the conversion.

***Table S2.*** *Optimization of the ammonium acetate loading.*

**
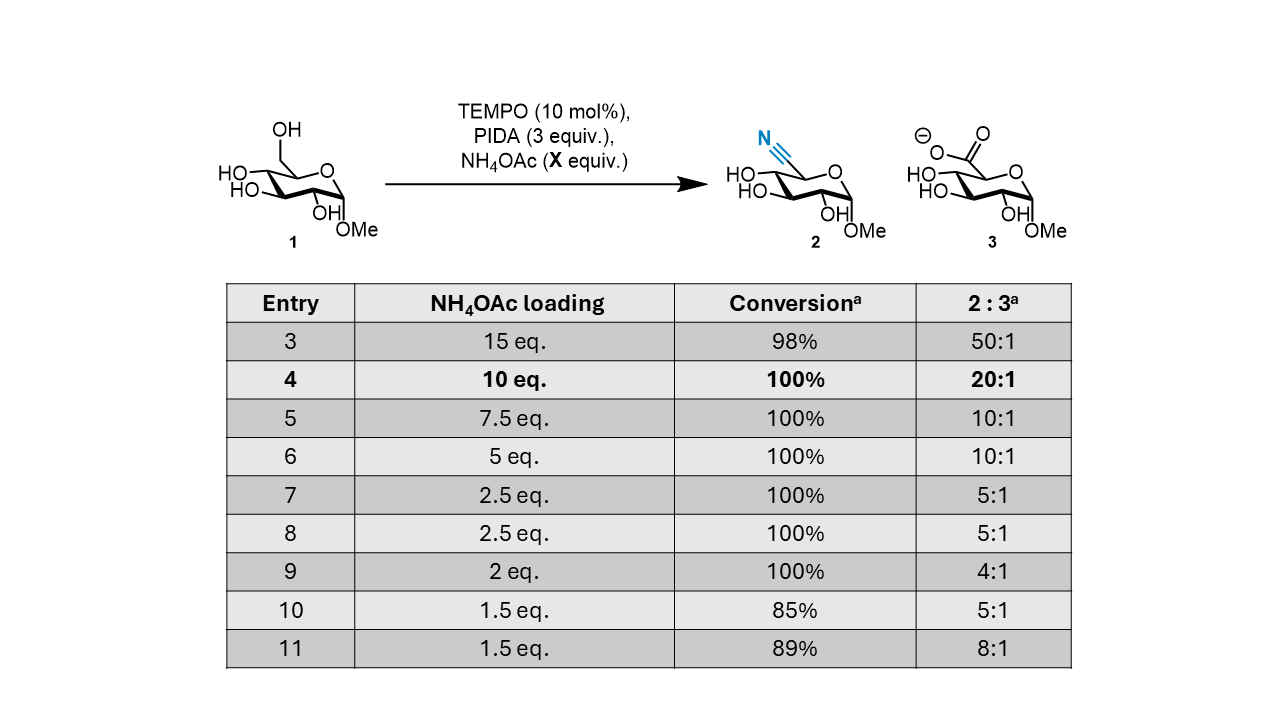
**

*Reactions were performed on 0.77 mmol scale, in 2 mL 1:1 (v/v) CH_3_CN/H_2_O. Stirring for 2 h at room temperature. ^a^As determined by crude ^1^H-NMR (400 MHz, CD_3_OD).*

## 4.3 PIDA loading optimization

The PIDA loading could be decreased from 3 eq. to 2.5 eq. without any impact on the reaction performance.

***Table S3.*** *Optimization of the PIDA loading.*


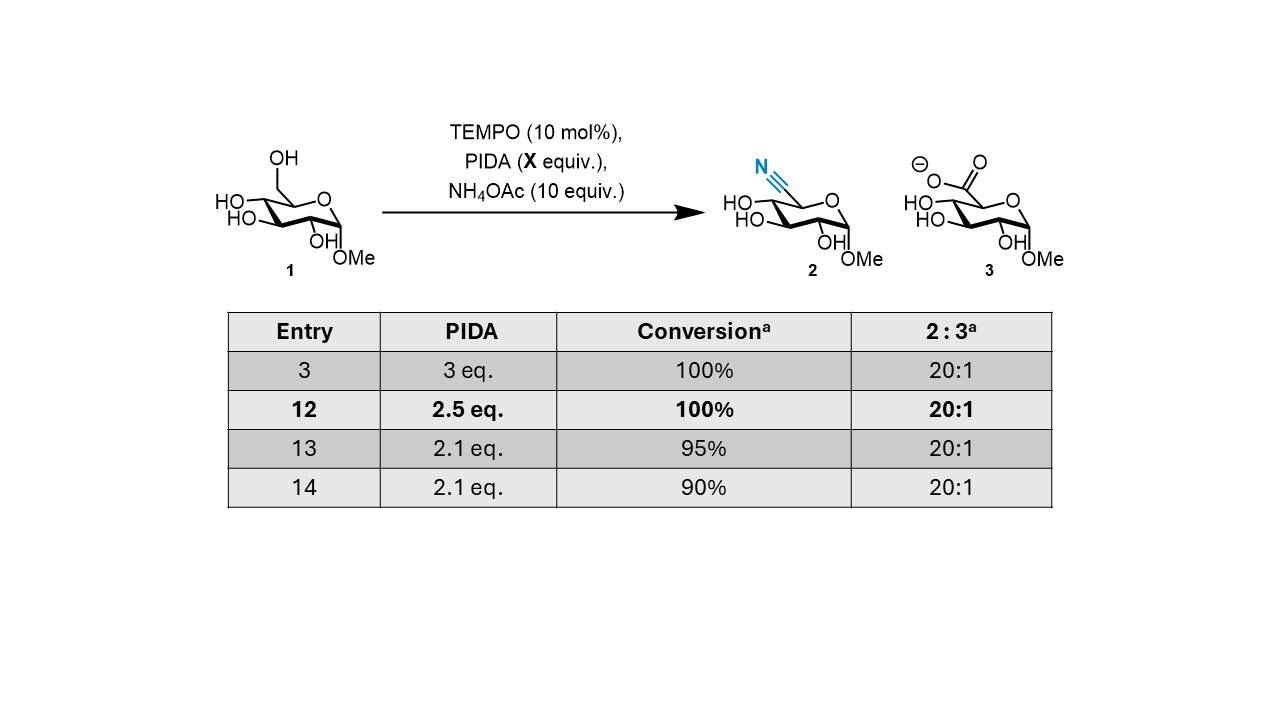


*Reactions were performed on 0.77 mmol scale, in 2 mL 1:1 (v/v) CH_3_CN/H_2_O. Stirring for 2 h at room temperature. ^a^As determined by crude ^1^H-NMR (400 MHz, CD_3_OD).*

## 4.4 Ammonium source optimization

Apart from ammonium acetate, other ammonium salts were screened. None of the attempted ammonium salts showed an improvement compared to ammonium acetate.

***Table S4.*** *Optimization of the ammonium source.*


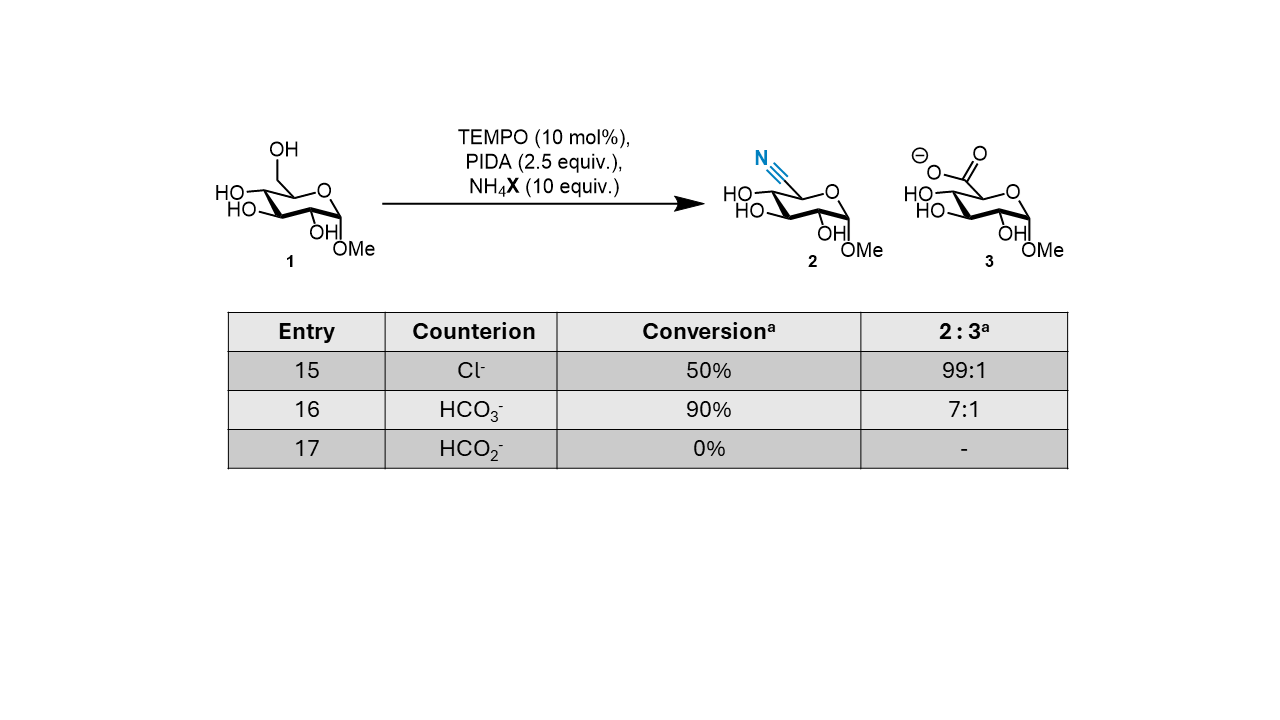


*Reactions were performed on 0.77 mmol scale, in 2 mL 1:1 (v/v) CH_3_CN/H_2_O. Stirring for 2 h at room temperature. ^a^As determined by crude ^1^H-NMR (400 MHz, CD_3_OD).*

# 5. Synthesis of Sugar Nitriles: Experimental Procedures and Characterization

**(2R,3S,4S,5R,6S)-3,4,5-trihydroxy-6-methoxytetrahydro-2H-pyran-2-carbonitrile** **(2)**

Prepared via the general procedure outlined in section 2. Isolated as an off-white very thick syrup (0.56 mmol, 106 mg, 73% yield).

**^1^H NMR (400 MHz, CD_3_OD)** δ 4.73 (d, *J* = 3.7 Hz, 1H), 4.35 (d, *J* = 9.8 Hz, 1H), 3.58 – 3.46 (m, 2H), 3.45 (s, 3H), 3.42 (dd, *J* = 9.4, 3.7 Hz, 1H); **^13^C NMR (101 MHz, CD_3_OD)** δ 118.66, 102.12, 74.19, 73.49, 72.67, 63.28, 56.55; **HRMS (ESI)** calculated for C_7_H_11_NO_5_NH_4_+ ([M+NH_4_]^+^): 207.0976, found: 207.0975; $\left[ \boldsymbol{\alpha} \right]_{\boldsymbol{D}}^{\boldsymbol{20}}\boldsymbol{:}$ +128 (*c* = 0.1, MeOH).

**(2R,3S,4S,5R,6R)-3,4,5-trihydroxy-6-methoxytetrahydro-2H-pyran-2-carbonitrile** (**4)**

Prepared via the general procedure outlined in section 2. Isolated as a white solid (0.56 mmol, 105 mg, 72% yield).

**^1^H NMR (400 MHz, CD_3_OD)** δ 4.26 – 4.23 (m, 2H, overlapping H1 and H5, coupling of the peaks can be determined manually for the individual protons: *J_H1,2_* = 7.7 Hz, *J_H4,5_* = 9.8 Hz), 3.52 (s, 3H), 3.51 – 3.43 (m, 1H), 3.35 – 3.27 (m, 2H), 3.17 (dd, *J* = 9.3, 7.7 Hz, 1H); **^13^C NMR (101 MHz, CD_3_OD)** δ 118.02, 105.87, 76.80, 74.32, 73.19, 66.81, 57.72. **HRMS (ESI)** calculated for C_7_H_11_NO_5_H+ ([M+H]^+^): 190.0710, found: 190.0711; $\left[ \boldsymbol{\alpha} \right]_{\boldsymbol{D}}^{\boldsymbol{20}}\boldsymbol{:}$ -82 (*c* = 0.1, MeOH); **m.p. (^o^C)**: 180 (decomp.)

**(2R,3S,4S,5R,6S)-3,4,5-trihydroxy-6-phenoxytetrahydro-2H-pyran-2-carbonitrile (5)**

Prepared via the general procedure outlined in section 2. Isolated as a white solid (0.56 mmol, 159 mg, 82% yield).

**^1^H NMR (400 MHz, CD_3_OD)** δ 7.34 – 7.26 (m, 2H), 7.12 – 7.00 (m, 3H), 5.00 (d, *J* = 6.2 Hz, 1H), 4.47 (d, *J* = 10.0 Hz, 1H), 3.66 – 3.58 (m, 1H), 3.54 – 3.42 (m, 2H); **^13^C NMR (101 MHz, CD_3_OD)** δ 158.41, 130.47, 123.95, 117.85, 102.24, 76.52, 74.07, 72.91, 66.60; **HRMS (ESI)** calculated for C_12_H_12_NO_5_- ([M−H]^−^): 250.0721, found: 250.0723.

**N-((2S,3R,4R,5S,6R)-6-cyano-4,5-dihydroxy-2-methoxytetrahydro-2H-pyran-3-yl)acetamide (6)**

Prepared via the general procedure outlined in section 2. Isolated as a white solid (0.35 mmol, 80 mg, 45% yield).

**^1^H NMR (400 MHz, CD_3_OD)** δ 4.74 – 4.71 (m, 1H), 4.41 – 4.33 (m, 1H), 3.94 (ddd, *J* = 9.0, 3.6, 1.2 Hz, 1H), 3.62 – 3.50 (m, 2H), 3.42 (s, 3H), 1.97 (s, 3H); **^13^C NMR (101 MHz, CD_3_OD)** δ 173.72, 118.51, 100.60, 73.84, 71.99, 63.37, 56.52, 54.60, 22.51; **HRMS (ESI)** calculated for C_9_H_14_N_2_O_5_H+ ([M+H]^+^): 231.0976, found: 231.0976; $\left[ \boldsymbol{\alpha} \right]_{\boldsymbol{D}}^{\boldsymbol{20}}\boldsymbol{:}$ +140 (*c* = 0.1, MeOH); **m.p. (^o^C)**: 205 (decomp.)

**Tert-butyl ((2S,3R,4R,5S,6R)-6-cyano-4,5-dihydroxy-2-methoxytetrahydro-2H-pyran-3-yl)carbamate (7)**

Prepared via the general procedure outlined in section 2. Isolated as a white solid (0.37 mmol, 107 mg, 48% yield).

**^1^H NMR (400 MHz, CD_3_OD)** δ 4.72 (d, *J* = 3.5 Hz, 1H), 4.35 (d, *J* = 9.3 Hz, 1H), 3.61 – 3.45 (m, 3H), 3.42 (s, 3H), 1.44 (s, 9H); **^13^C NMR (101 MHz, CD_3_OD)** δ 157.76, 118.13, 100.70, 80.07, 73.46, 71.81, 62.94, 56.14, 55.55, 28.26; **HRMS (ESI)** calculated for C_12_H_19_N_2_O_6_- ([M−H]^−^): 287.1249, found: 287.1251; $\left[ \boldsymbol{\alpha} \right]_{\boldsymbol{D}}^{\boldsymbol{20}}\boldsymbol{:}$ +98 (*c* = 0.1, MeOH); **m.p. (^o^C)**: 75

**N-((2S,3R,4R,5S,6R)-6-cyano-4,5-dihydroxy-2-isopropoxytetrahydro-2H-pyran-3-yl)acetamide (8)**

Prepared via the general procedure outlined in section 2. Isolated as a white solid (0.39 mmol, 100 mg, 50% yield).

**^1^H NMR (400 MHz, CD_3_OD)** δ 4.97 (d, *J* = 3.8 Hz, 1H), 4.44 (d, *J* = 9.8 Hz, 1H), 3.93 – 3.83 (m, 2H), 3.62 – 3.49 (m, 2H), 1.97 (s, 3H), 1.25 (d, *J* = 6.2 Hz, 3H), 1.15 (d, *J* = 6.1 Hz, 3H); **^13^C NMR (101 MHz, CD_3_OD)** δ 173.23, 118.16, 97.13, 73.64, 72.33, 71.33, 63.12, 54.41, 23.01, 21.98, 21.26; **HRMS (ESI)** calculated for C_11_H_18_N_2_O_5_H+ ([M+H]^+^): 259.1289, found: 259.1286; $\left[ \boldsymbol{\alpha} \right]_{\boldsymbol{D}}^{\boldsymbol{20}}\boldsymbol{:}$ +156 (*c* = 0.1, MeOH); **m.p. (^o^C)**: 213 (decomp.).

**Benzyl ((2S,3R,4R,5S,6R)-6-cyano-4,5-dihydroxy-2-methoxytetrahydro-2H-pyran-3-yl)carbamate (9)**

Prepared via the general procedure outlined in section 2. Isolated as a white solid (0.45 mmol, 144 mg, 58% yield).

**^1^H NMR (400 MHz, CD_3_OD)** δ 7.45 – 7.22 (m, 5H), 5.08 (s, 2H), 4.74 (d, *J* = 3.5 Hz, 1H), 4.41 – 4.31 (m, 1H), 3.72 – 3.60 (m, 1H), 3.60 – 3.49 (m, 2H), 3.41 (s, 3H); **^13^C NMR (101 MHz, CD_3_OD)** δ 158.76, 138.17, 129.43, 128.99, 128.89, 118.53, 100.99, 73.83, 72.25, 67.66, 63.34, 56.56, 56.45; **HRMS (ESI)** calculated for C_15_H_18_N_2_O_6_Na ([M+Na]^+^): 345.1063, found: 345.1058; $\left[ \boldsymbol{\alpha} \right]_{\boldsymbol{D}}^{\boldsymbol{20}}\boldsymbol{:}$ +84 (*c* = 0.1, MeOH); **m.p. (^o^C)**: 166.

**(2R,3S,4R,6S)-3,4-dihydroxy-6-isopropoxytetrahydro-2H-pyran-2-carbonitrile (10)**

Prepared from an α, β mixture of isopropyl 2-deoxyglucoside via the general procedure outlined in section 2. Mixture of epimers isolated as a brown oil (0.45 mmol, 91 mg, 58% yield).

**^1^H NMR (400 MHz, CD_3_OD)** δ 5.08 (d, *J* = 3.7 Hz, 1H), 4.36 (d, *J* = 10.0 Hz, 1H), 3.89 (p, *J* = 6.2 Hz, 1H), 3.80 – 3.70 (m, 1H), 3.44 – 3.36 (m, 1H), 1.98 (dd, *J* = 12.4, 5.8 Hz, 1H), 1.70 – 1.58 (m, 1H), 1.21 (d, *J* = 6.25 Hz, 3H), 1.15 (d, *J* = 6.1 Hz, 3H); **^13^C NMR (101 MHz, CD_3_OD)** δ 118.20, 97.00, 73.85, 70.61, 68.27, 63.22, 37.98, 22.94; **HRMS (ESI)** calculated for C_9_H_15_NO_4_H+ ([M+H]^+^): 202.1074, found: 202.1073.

**(2R,3R,4S,5R,6S)-3,4,5-trihydroxy-6-methoxytetrahydro-2H-pyran-2-carbonitrile (11)**

Prepared via the general procedure outlined in section 2. Isolated as a white solid (0.54 mmol, 102 mg, 70% yield).

**^1^H NMR (400 MHz, CD_3_OD)** δ 4.82 (dd, *J* = 1.6, 0.7 Hz, 1H), 4.77 (d, *J* = 3.3 Hz, 1H), 4.01 (dd, *J* = 3.3, 1.9 Hz, 1H), 3.75 (qd, *J* = 10.0, 3.3 Hz, 2H), 3.45 (s, 3H); **^13^C NMR (101 MHz, CD_3_OD)** δ 117.34, 105.98, 73.43, 71.48, 70.56, 66.46, 57.58; **HRMS (ESI)** calculated for C_7_H_10_NO_5_- ([M−H]^−^): 188.0565, found: 188.0565; $\left[ \boldsymbol{\alpha} \right]_{\boldsymbol{D}}^{\boldsymbol{20}}\boldsymbol{:}$ +170 (*c* = 0.1, MeOH); **m.p. (^o^C)**: 173.

**(2R,3R,4S,5R,6S)-3,4,5-trihydroxy-6-methoxytetrahydro-2H-pyran-2-carbonitrile (12)**

Prepared via the general procedure outlined in section 2. Isolated as a white solid (0.54 mmol, 102 mg, 70% yield).

**^1^H NMR (400 MHz, CD_3_OD)** δ 4.69 (d, *J* = 2.0 Hz, 1H), 4.29 (d, *J* = 9.9 Hz, 1H), 3.86 (t, *J* = 9.6 Hz, 1H), 3.79 (dd, *J* = 3.3, 1.9 Hz, 1H), 3.59 (dd, *J* = 9.3, 3.3 Hz, 1H), 3.42 (s, 3H); **^13^C NMR (101 MHz, CD_3_OD)** δ 118.69, 103.63, 71.64, 71.30, 69.77, 64.09, 56.14; **HRMS (ESI)** calculated for C_7_H_10_NO_5_- ([M−H]^−^): 188.0565, found: 188.0565.

**(2R,3R,4S,5R,6R)-3,4,5-trihydroxy-6-methoxytetrahydro-2H-pyran-2-carbonitrile (13)**

Prepared via the general procedure outlined in section 2. Isolated as a white solid (0.56 mmol, 105 mg, 72% yield).

**^1^H NMR (400 MHz, CD_3_OD)** δ 4.68 (d, *J* = 1.6 Hz, 1H), 4.19 (d, *J* = 5.2 Hz, 1H), 3.97 – 3.94 (m, 1H), 3.56 – 3.49 (m, 5H); **^13^C NMR (101 MHz, CD_3_OD)** δ 117.34, 105.98, 73.43, 71.48, 70.56, 66.46, 57.58; **HRMS (ESI)** calculated for C_7_H_10_NO_5_- ([M−H]^−^): 188.0565, found: 188.0566; $\left[ \boldsymbol{\alpha} \right]_{\boldsymbol{D}}^{\boldsymbol{20}}\boldsymbol{:}$ -66 (*c* = 0.1, MeOH); **m.p. (^o^C)**: 170.

**(3aR,5R,6S,6aR)-6-hydroxy-2,2-dimethyltetrahydrofuro[2,3-d][1,3]dioxole-5-carbonitrile (17)**

To a pressure tube equipped with a stirring bar was added PIDA (914 mg, 2.84 mmol, 2.5 eq.), TEMPO (18 mg, 0.11 mmol, 10 mol%), ammonium acetate (525 mg, 6.81 mmol, 6 eq.) and 1,2-*O*-isopropylidene-ɑ-D-glucofuranose (250 mg, 1.14 mmol, 1 eq.). Acetonitrile (3 mL) and water (1 mL) were added to the mixture resulting in a biphasic system. The mixture was allowed to stir for 2 h at room temperature and subsequently purified by flash column chromatography (10% MeOH in DCM). The pure product was obtained as a brown oil (0.59 mmol, 110 mg, 52% yield) and characterized by ^1^H-NMR, ^13^C-NMR and HRMS.

**^1^H-NMR (400 MHz, CD_3_OD)** δ 5.96 (d, J = 3.4 Hz, 1H), 4.94 (d, J = 3.0 Hz, 1H), 4.55 (d, J = 3.4 Hz, 1H), 4.34 (d, J = 4.4 Hz, 0H), 1.45 (s, 2H), 1.30 (s, 1H); **^13^C-NMR (101 MHz, CD_3_OD)** δ 116.50, 113.87, 106.92, 85.79, 76.36, 72.19, 27.16, 26.28; **HRMS (APCI)** calculated for C_8_H_11_NO_4_H+ ([M+H]^+^): 186.0761, found: 186.0762.

**(3aR,5R,6R,6aR)-6-hydroxy-2,2-dimethyltetrahydrofuro[2,3-d][1,3]dioxole-5-carbonitrile (19)**

To a pressure tube equipped with a stirring bar was added PIDA (914 mg, 2.84 mmol, 2.5 eq.), TEMPO (18 mg, 0.11 mmol, 10 mol%), ammonium acetate (525 mg, 6.81 mmol, 6 eq.) and 1,2-O-isopropylidene-ɑ-D-allofuranose (250 mg, 1.14 mmol, 1 eq.). Acetonitrile (3 mL) and water (1 mL) were added to the mixture resulting in a biphasic system. The mixture was allowed to stir for 2 h at room temperature and subsequently purified by flash column chromatography (10% MeOH in DCM). The pure product was obtained as a brown oil (0.62 mmol, 115 mg, 45% yield) and characterized by ^1^H-NMR, ^13^C-NMR and HRMS.

**^1^H-NMR (400 MHz, CD_3_OD)** δ 5.80 (d, J = 3.4 Hz, 1H), 4.59 (t, J = 3.9 Hz, 1H), 4.48 (d, J = 9.1 Hz, 1H), 4.29 (dd, J = 9.1, 4.3 Hz, 1H), 1.50 (s, 3H), 1.32 (s, 3H); **^13^C-NMR (101 MHz, CD_3_OD)** δ 117.15, 113.38, 104.88, 78.56, 75.00, 67.33, 48.23, 48.02, 47.80, 47.59, 47.38, 47.16, 46.95, 25.51, 25.02; **HRMS (APCI)** calculated for C_8_H_11_NO_4_H+ ([M+H]^+^): 186.0761, found: 186.0760.

# 6. Synthesis of Sugar Nitrile Derivatives: Experimental Procedures and Characterization

**(2S,3R,4S,5S,6R)-2-methoxy-6-(6-methyl-1,2,4,5-tetrazin-3-yl)tetrahydro-2H-pyran-3,4,5-triol (22)**

To a 10 mL reaction vial equipped with a stir bar, **2** (112 mg, 0.59 mmol, 1 eq.), acetonitrile (0.4 mL, 7.62 mmol, 13 eq.), 3-mercaptopropionic acid (35 μL, 0.40 mmol, 0.7 eq.), hydrazine hydrate (0.8 mL, 16.49 mmol, 28 eq.) and ethanol (0.2 mL) were added. Under nitrogen, the reaction was stirred overnight at 40 °C. The reaction was concentrated *in vacuo* and the mixture was dissolved in an aqueous solution of sodium nitrite (1.04 g, 15.0 mmol, 26 eq.) and cooled to 0 °C. The mixture was acidified to pH 3 with 1M HCl after which a pink color was observed. The excess acid was neutralized using an aqueous sodium bicarbonate solution and the mixture was concentrated *in vacuo*. The crude product was then dissolved in acetonitrile (20 mL) and the remaining precipitate was filtered off. The filtrate was concentrated *in vacuo*. The resulting product was isolated as a bright pink oil (0.25 mmol, 65 mg , 42%) and was characterized by ^1^H-NMR, ^13^C-NMR and HRMS.

**^1^H-NMR (400 MHz, CD_3_OD)** δ 5.06 (d, J 10.0 Hz, 1H), 4.83 (d, *J* = 3.7 Hz, 1H), 4.08 (t, *J* = 9.0 Hz, 1H), 3.87 (t, *J* = 9.3 Hz, 1H), 3.66 (dd, *J* = 9.6, 3.7 Hz, 1H), 3.50 (s, 3H), 3.05 (s, 3H); **^13^C-NMR (101 MHz, CD_3_OD)** δ 170.45, 167.65, 102.35, 74.75, 74.31, 73.99, 73.43, 56.08, 21.27; **HRMS (ESI)** calculated for C9H14N4O5Na+ ([M+Na]^+^): 281.0856, found: 281.0858; $\left[ \boldsymbol{\alpha} \right]_{\boldsymbol{D}}^{\boldsymbol{20}}\boldsymbol{:}$ +60 (*c* = 0.1, MeOH).

**(2S,3S,4S,5R,6S)-2-(benzo[d]thiazol-2-yl)-6-methoxytetrahydro-2H-pyran-3,4,5-triol (23)**

A 50 mL round-bottom flask equipped with a stir bar was placed under inert atmosphere and charged with **2** (600 mg, 3.17 mmol, 1 eq.), 2-aminothiophenol (337 μL, 3.17 mmol, 1 eq.) and potassium bicarbonate (318 mg, 3.172 mmol, 1 eq.) in methanol (10 mL). The mixture was stirred at 40 ^o^C. After 16 h the reaction went to full completion, as determined by TLC (10% MeOH in DCM). The reaction mixture was extracted with DCM and washed with water before drying over brine and MgSO_4_. The crude was further purified by flash column chromatography (10% MeOH in DCM) and obtained as a white solid (2.85 mmol, 847 mg, 90%).

**^1^H-NMR (400 MHz, CD_3_OD)** δ 8.00 (dt, J = 7.9, 1.7 Hz, 2H), 7.52 (ddd, J = 8.4, 7.2, 1.3 Hz, 1H), 7.45 (ddd, J = 8.3, 7.3, 1.2 Hz, 1H), 4.93 – 4.86 (m, 2H), 3.81 (dd, J = 9.7, 8.8 Hz, 1H), 3.65 – 3.57 (m, 2H), 3.50 (s, 3H); **^13^C-NMR (101 MHz, CDCl_3_)** δ 170.78, 152.44, 134.41, 126.26, 125.39, 123.19, 121.77, 100.33, 74.30, 73.45, 71.70, 71.13, 56.03; **HRMS (ESI)** calculated for C_13_H_15_NO_5_SNa+ ([M+Na]^+^): 320.0563, found: 320.0561; $\left[ \boldsymbol{\alpha} \right]_{\boldsymbol{D}}^{\boldsymbol{20}}\boldsymbol{:}$ +108 (*c* = 0.1, MeOH); **m.p. (^o^C)**: 135.

**(2R,3S,4S,5R,6S,Z)-N',3,4,5-tetrahydroxy-6-methoxytetrahydro-2H-pyran-2-carboximidamide (24)**

A flask equipped with a stirring bar and **2** (200 mg, 1.06 mmol) was heated to 47 °C. Using a syringe, 50 wt% aqueous hydroxylamine (1 mL) was added dropwise. The reaction was stirred overnight. After completion, the reaction mixture was concentrated *in vacuo*. The crude product was dissolved in methanol (1 mL), and the addition of acetone (10 mL) caused a precipitate to form. The precipitate was filtered off and washed with acetone. The product was obtained as a very thick syrup (1.00 mmol, 240 mg, 53%) and characterized by ^1^H-NMR, ^13^C-NMR and HRMS.

**^1^H-NMR (400 MHz, CD_3_OD)** δ 4.71 (d, *J* = 3.7 Hz, 1H), 3.88 (d, *J* = 9.8 Hz, 1H), 3.63 (t, *J* = 9.5 Hz, 1H), 3.54 (t, *J* = 9.7 Hz, 1H), 3.48 (dd, *J* = 9.5, 3.8 Hz, 1H), 3.42 (s, 3H);**^13^C-NMR (101 MHz, CD_3_OD)** δ 152.89, 100.25, 73.21, 71.78, 71.53, 70.38, 54.51; **HRMS (APCI)** calculated for C_7_H_14_N_2_O_6_H+ ([M+H]^+^): 223.0925, found: 223.0921; $\left[ \boldsymbol{\alpha} \right]_{\boldsymbol{D}}^{\boldsymbol{20}}\boldsymbol{:}$ +126 (*c* = 0.1, MeOH).

**(R)-2-((2S,3S,4S,5R,6S)-3,4,5-trihydroxy-6-methoxytetrahydro-2H-pyran-2-yl)-4,5-dihydrothiazole-4-carboxylic acid (25)**

A 50 mL round-bottom flask equipped with a stir bar was placed under inert atmosphere and charged with **2** (225 mg, 1.19 mmol, 1 eq.), L-cysteine (216 mg, 1.78 mmol, 1.5 eq.) and sodium bicarbonate (400 mg, 4.76 mmol, 4 eq.) in N_2_-sparged ethanol (10 mL). The mixture was stirred at reflux for 35 h. All volatiles were evaporated and acetone (10 mL) was added to the crude solid. The suspension was sonicated and the supernatant was removed. This procedure was repeated twice and the precipitate was concentrated under reduced pressure to afford the title compound as an off-white solid. The purity of the product was determined by qNMR to be 45 mass%, with bicarbonate salts being present. The qNMR-corrected yield was found to be 76%.

**^1^H-NMR (400 MHz, D_2_O)** δ 5.01 (t, J = 9.5 Hz, 1H), 4.88 (d, J = 3.6 Hz, 1H), 4.48 (d, J = 9.8 Hz, 1H), 3.79 – 3.59 (m, 3H), 3.49 - 3.44 (m, 4H), 3.04 (m, 1H); **^13^C-NMR (101 MHz, D_2_O)** δ 178.01, 172.74, 99.68, 79.50, 72.28, 72.06, 70.93, 70.76, 55.46, 35.52; **HRMS (ESI)** calculated for C_10_H_15_NO_7_SNa+ ([M+Na]^+^): 316.0462, found: 316.0462.

**(2S,3R,4S,5S,6R)-2-methoxy-6-(5-phenyl-4,5-dihydro-1,2,4,5-oxadiazaborol-3-yl)tetrahydro-2H-pyran-3,4,5-triol (26)**

To an NMR-tube were added **24** (25.0 mg, 0.113 mmol, 1 eq.) and phenylboronic acid (14.7 mg, 0.121 mmol, 1.1 eq.) in DMSO-d6. The reaction was followed by ^1^H-NMR. An equilibrium was established within 30 min. An additional equivalent of boronic acid (15.0 mg, 0.123 mmol, 1.1 eq.) was added. Another equilibrium was established within 10 min after addition. The crude product was analyzed by ^1^H-NMR and ^13^C-NMR and the conversion was calculated to be 88%. No purification was attempted due to the instable nature of the product.

**^1^H-NMR (400 MHz, DMSO-d_6_)** δ 10.17 (s, 1H), 7.85 (m, 2H), 7.47 (m, 3H), 4.68 (d, J = 3.7 Hz, 1H), 4.35 (d, J = 9.7 Hz, 1H), 3.60 (t, J = 9.1 Hz, 1H), 3.54 (t, J = 9.0 Hz, 1H), 3.44 (dd, J = 9.3 Hz, 1H), 3.33 (s, 3H); **^13^C-NMR (101 MHz, DMSO-d_6_)** δ 159.86, 133.96, 131.21, 128.43, 100.65, 73.11, 71.77, 71.70, 66.60, 55.16.

# 7. Identification of C5-epimer

In most reactions, approximately 5% of a side-product was observed. TOCSY NMR of a sample with a reasonably large amount of this side product revealed its NMR correlations (Figure S1). The coupling patterns of these correlations were found to be consistent with those expected for a glycoside with an axial substituent at the C5-position (Figure S2). It is important to note that, in this case, this side-product is observed in the complete absence of the carboxylic acid side product, suggesting that this side-product is the C5-epimerized nitrile product.


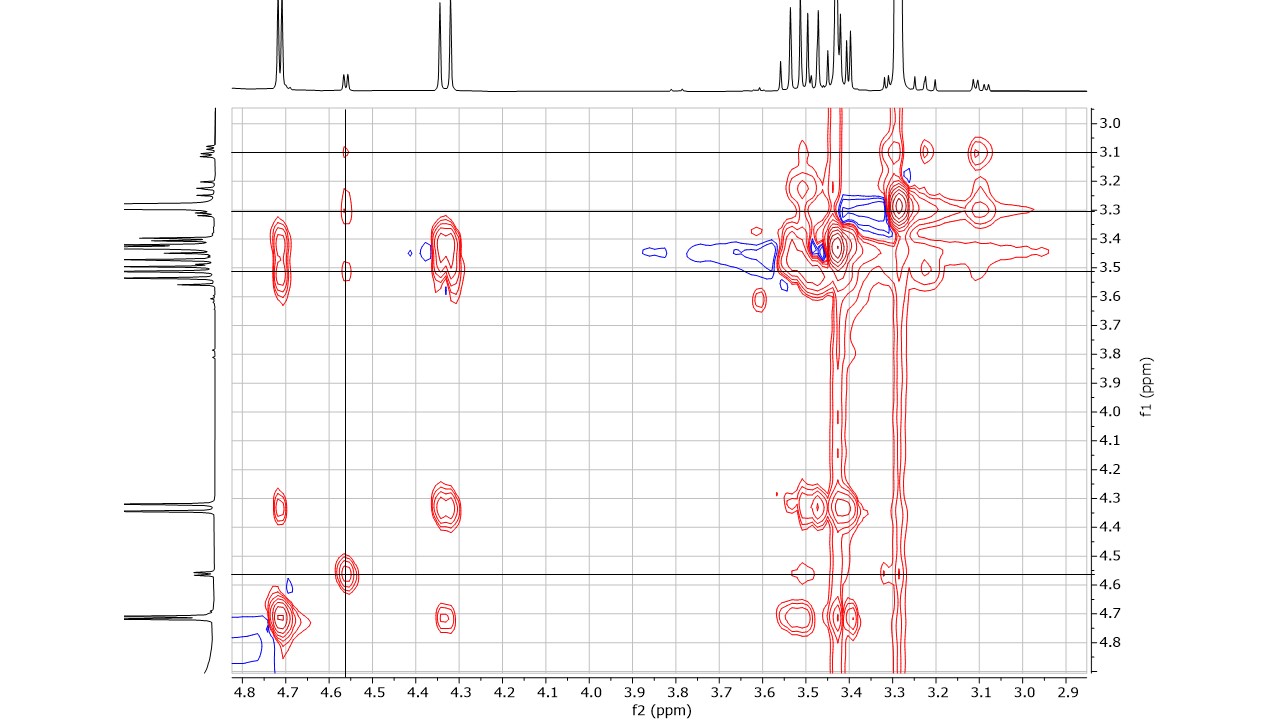


Figure S1 TOCSY of a crude ^1^H-NMR spectrum of the ammoxidation of α-methylglucopyranoside highlighting the correlations of the formed C5-epimer.


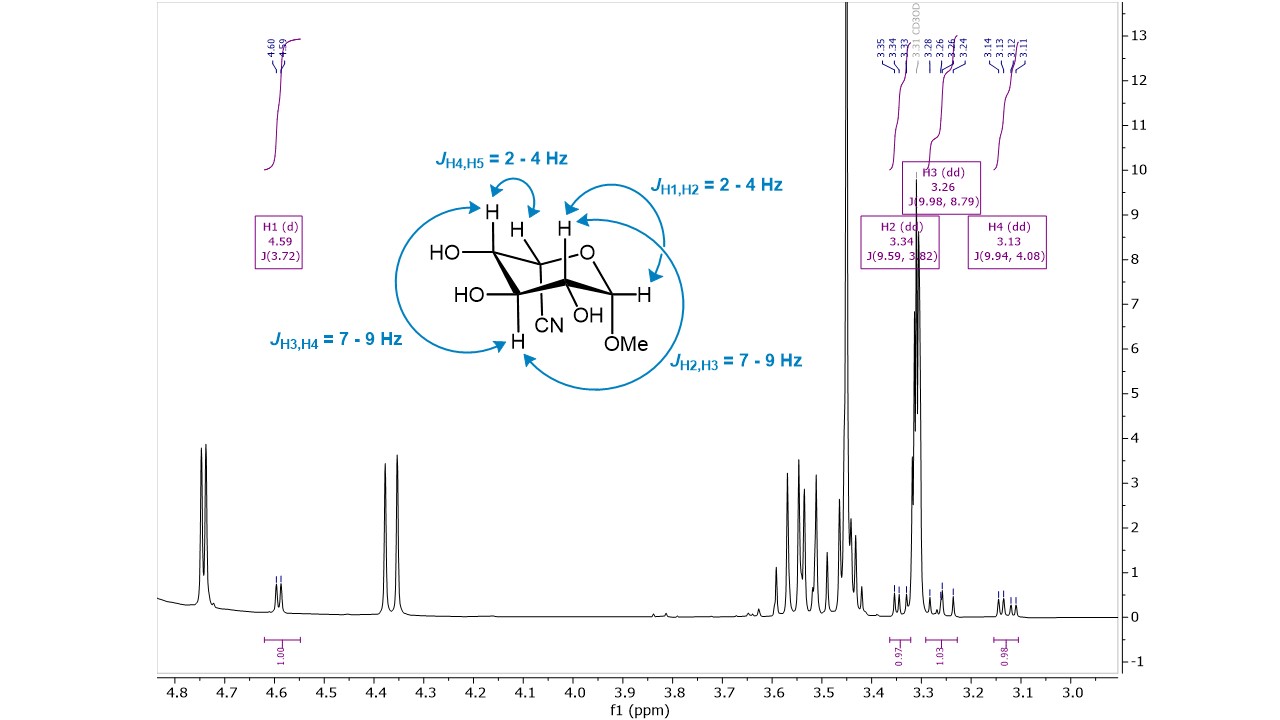


Figure S2 Part of a crude ^1^H-NMR spectrum of the ammoxidation of α-methylglucopyranoside highlighting the peaks of the formed C5-epimer. Inset shows the expected ^1^H-NMR coupling patterns of C5-epimer.

This epimerization can be explained from the proposed intermediate **29** (Scheme S1). We expect the proton on the C5-position to have an increased acidity after formation of the phenyliodonium imine. Deprotonation at this position would lead to enamine **30** and restore the ylid. Formation of this enamine causes a loss of stereochemistry at the C5-position. Protonation of the enamine to afford the stereoinverted phenyliodonium imine **31** then leads to elimination and yields the C5-epimerized nitrile **32**.

Scheme S1 Proposed mechanism of the formation of the C5-epimerized nitrile product from the ammoxidation reaction.

# 8. MCR analysis of Raman spectral data

## 8.1 MCR analysis of Raman spectral data between 400-700 cm^-1^

The singular variant decomposition (SVD) analysis (Figure S4) shows that three components resolve (3.7-0.3)/3.7 = 92% of the spectral data (Figure S3). MCR analysis was carried out with four components for comparison and accounted for 99% of the data. The results of MCR analysis with three components (Figures S5, S6) shows components which are likely weighted combinations of the real spectra of the compounds present in solution. Nevertheless, reaction progress shows that in addition to starting material and final product, a clear third component that represents largely an intermediate species achieves significant concentrations during the reaction.

With four components (Figures S7, S8), the fourth component resembles the other three components (spectra) and, more importantly, shows a similar reaction progress as the third component indicating that the data is best fit with three components.


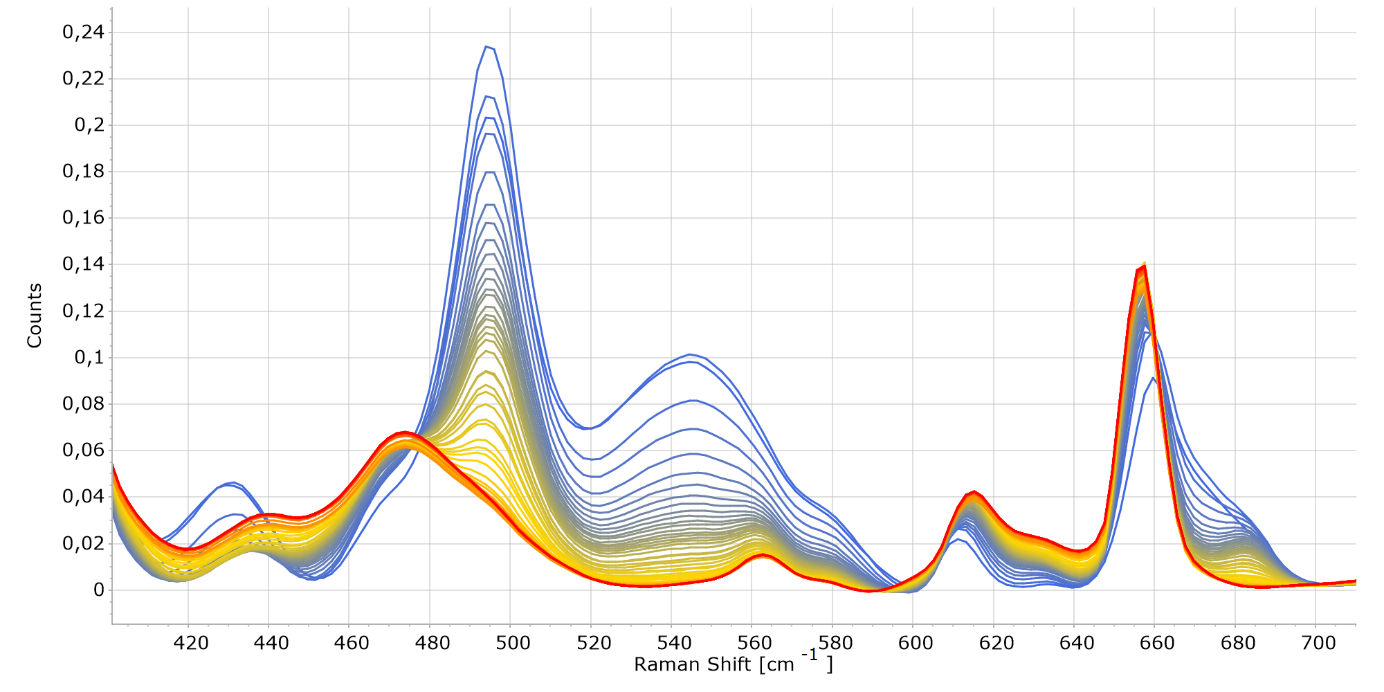


Figure S3. Data set used for MCR analysis


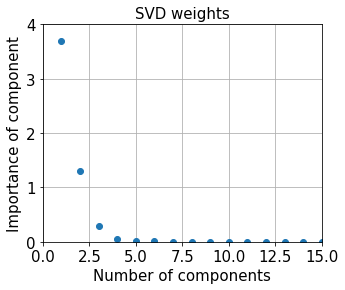


Figure S4. Outcome of SVD analysis.


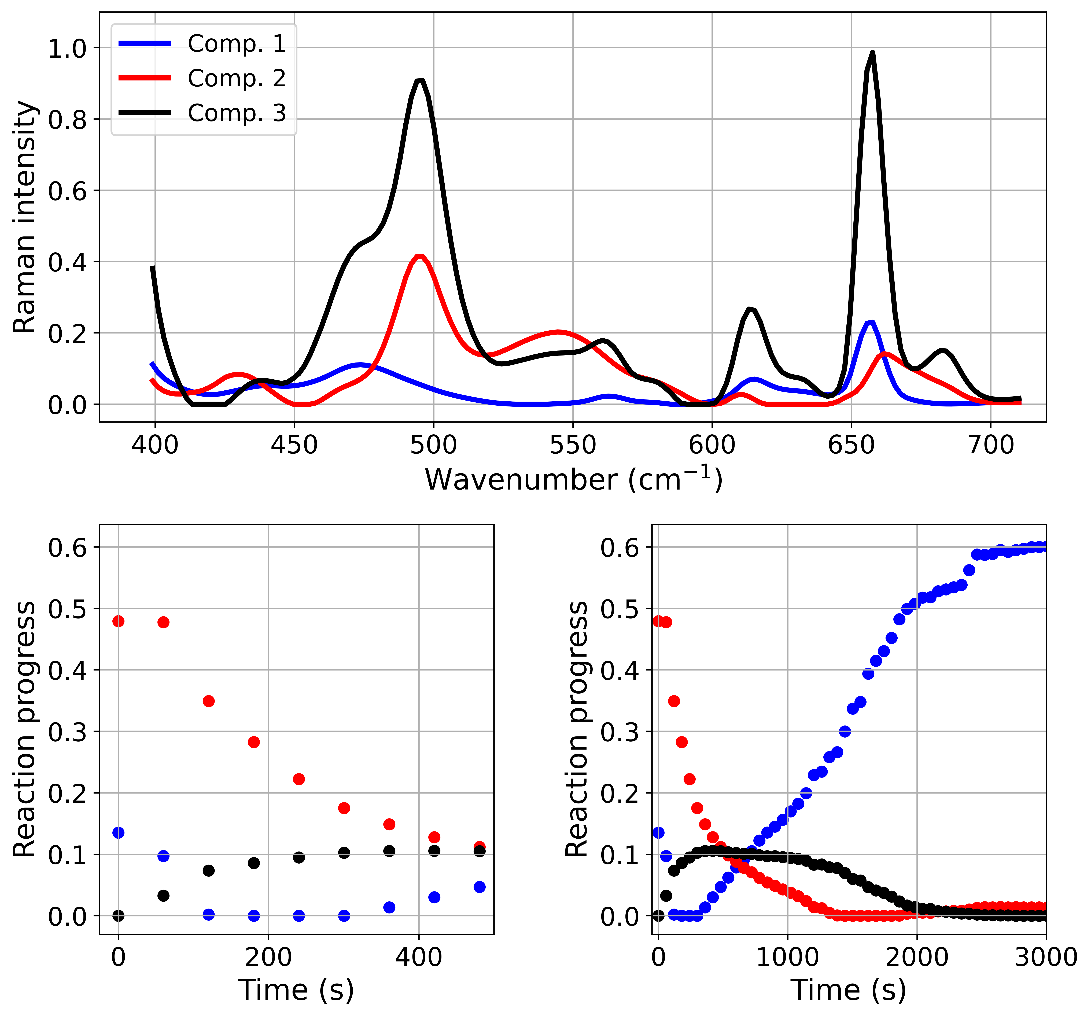


Figure S5. MCR analysis of data with three components.


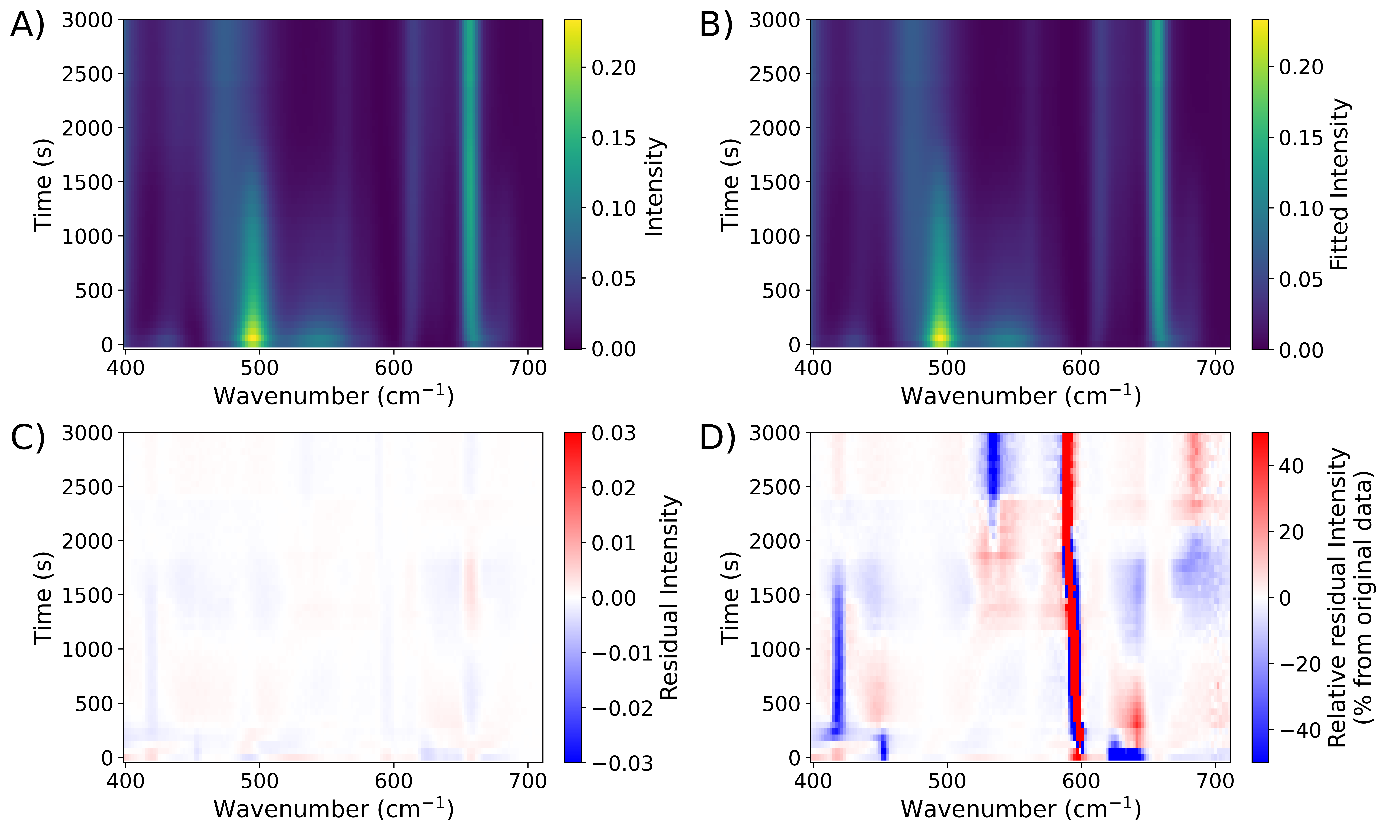


Figure S6. Error analysis. MCR analysis with three components.


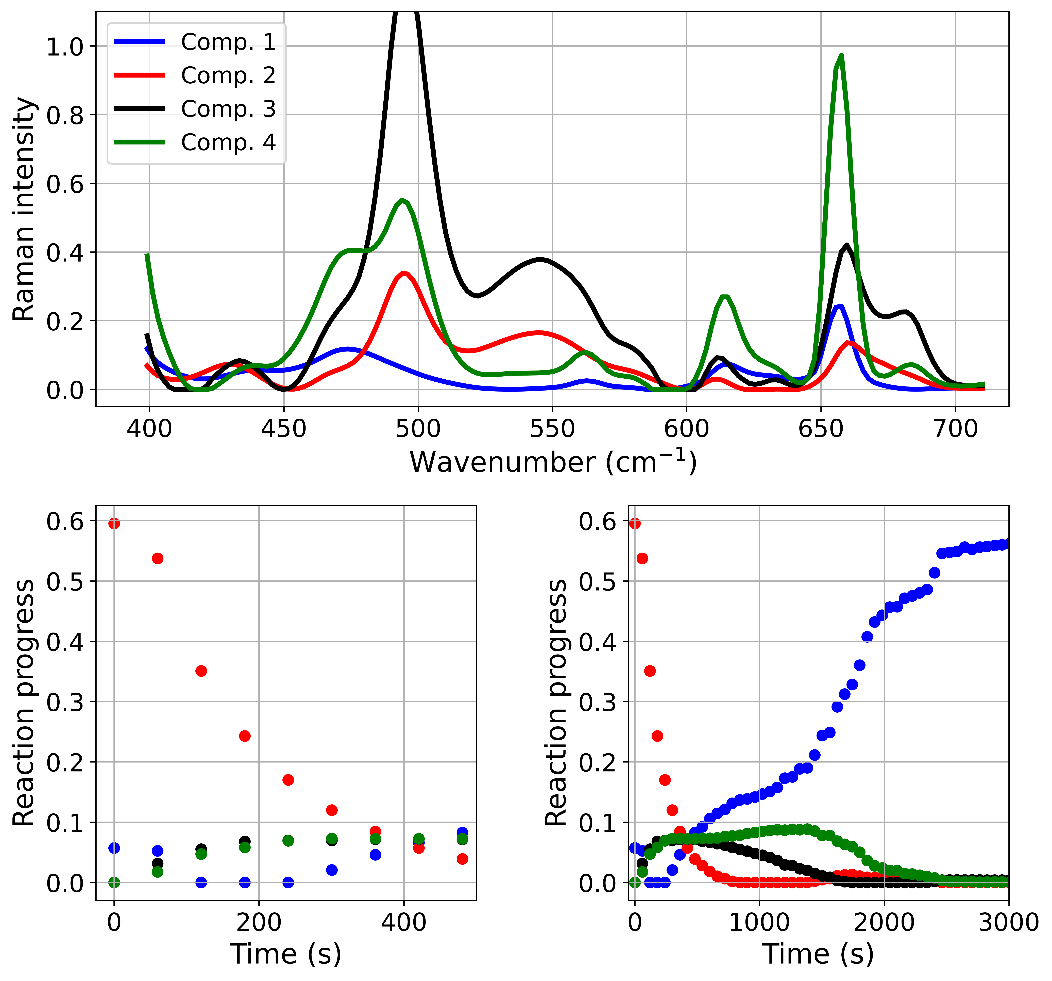


Figure S7. MCR analysis with four components. The reaction progress is similar for the third and the fourth components.


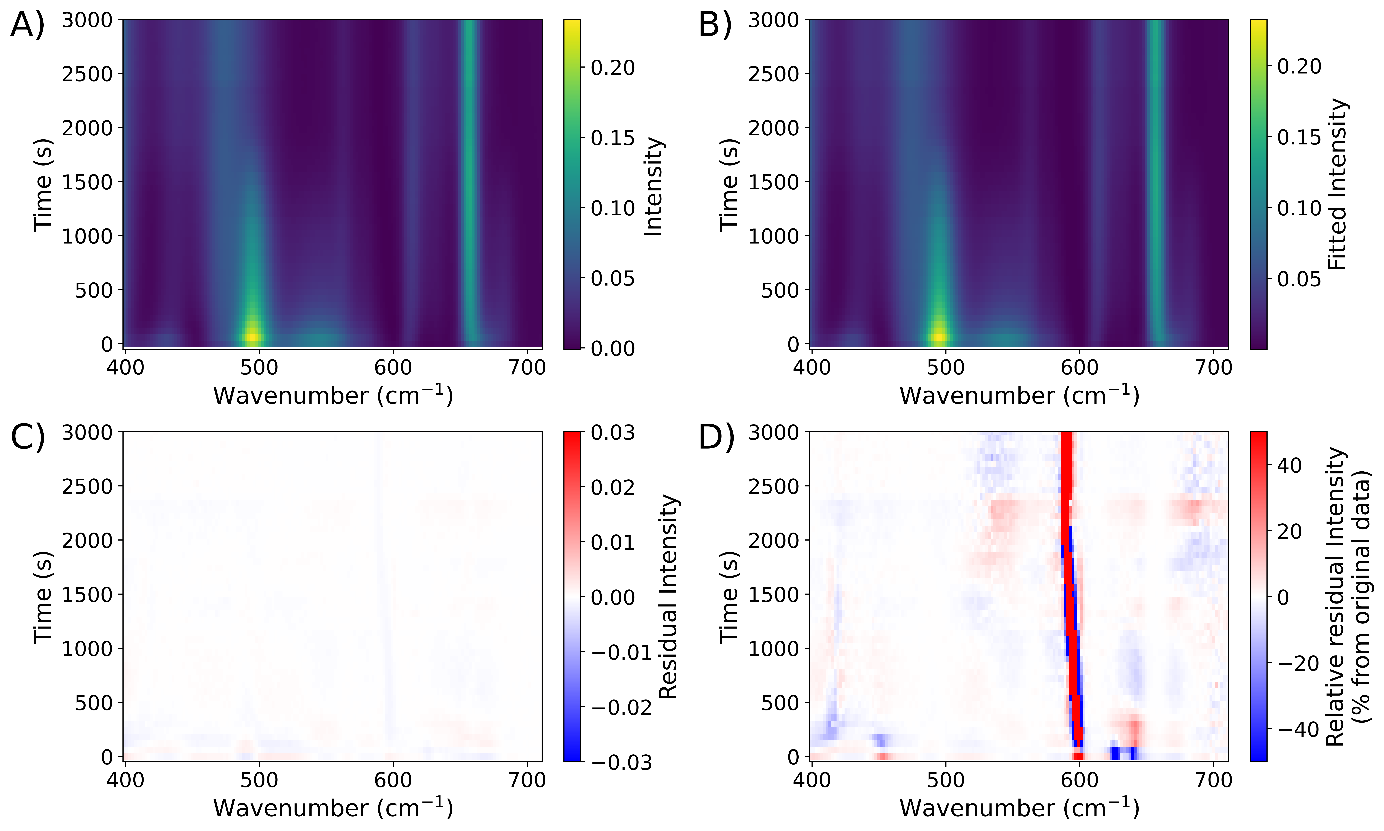


Figure S8. Error analysis with four components. The reaction progress is similar for the third and the fourth shape/spectrum.

## 8.2 MCR analysis of the spectral data between 975-1900 cm^-1^

SVD analysis (Figure S10) shows that the data can be described using two or three components (Figure S9). Two components resolve ((11-0.6)/11) = 95% of the data, while with 3 components 97.5% of the data is resolved, i.e. only a minor improvement in fitting.

MCR analysis with three components (Figures S11 and S12) shows that the third component overlaps greatly with either the first or the second component. Moreover, this component is not significant towards describing reaction progress. Therefore, the MCR analysis with two components (Figures S13 and S14) shows that this dataset can be resolved with two components, the substrate and the product of the reaction. The absence of the intermediate species (i.e., a third component in the MCR analysis) is consistent with the absence of iodine-related bands in this wavenumber range.


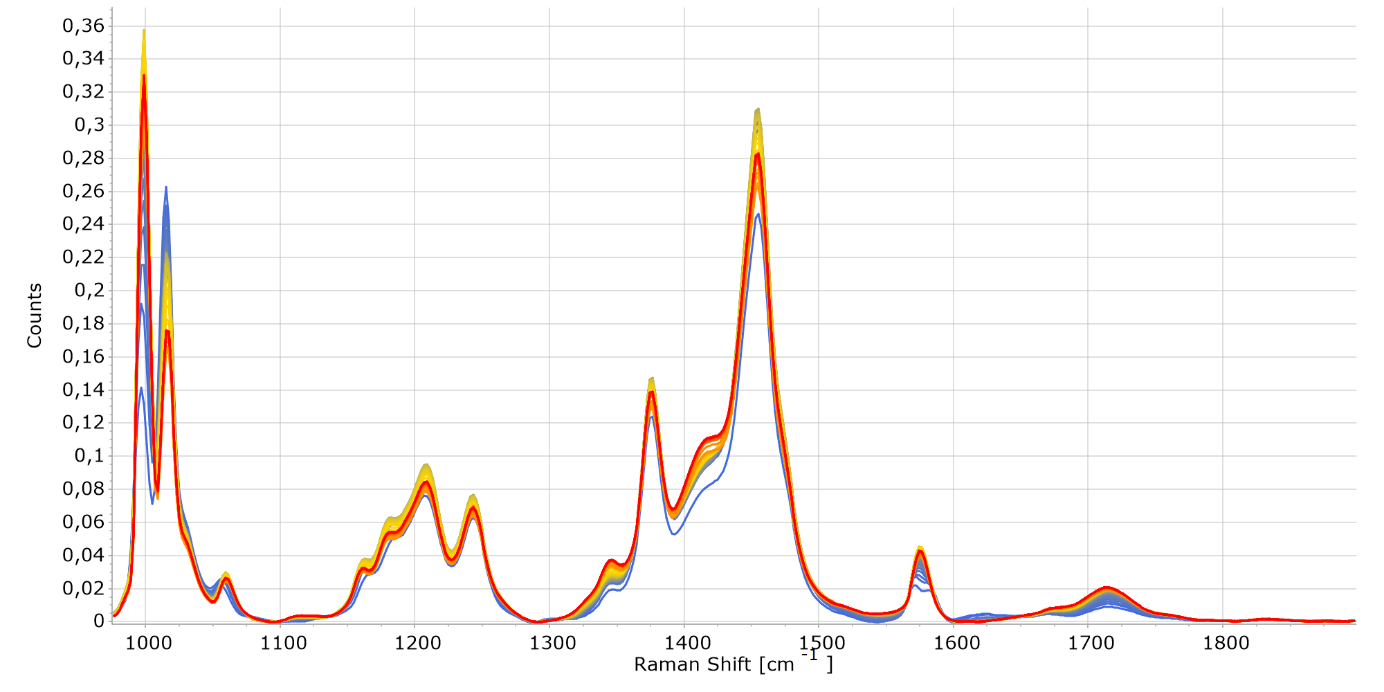


Figure S9. Raw spectral data in the range of 980-1900 cm^-1^.


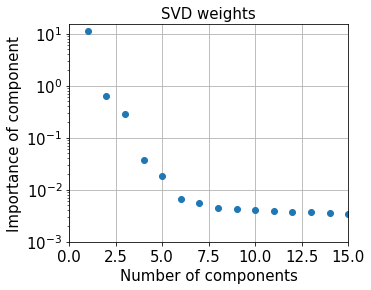


Figure S10. SVD analysis.


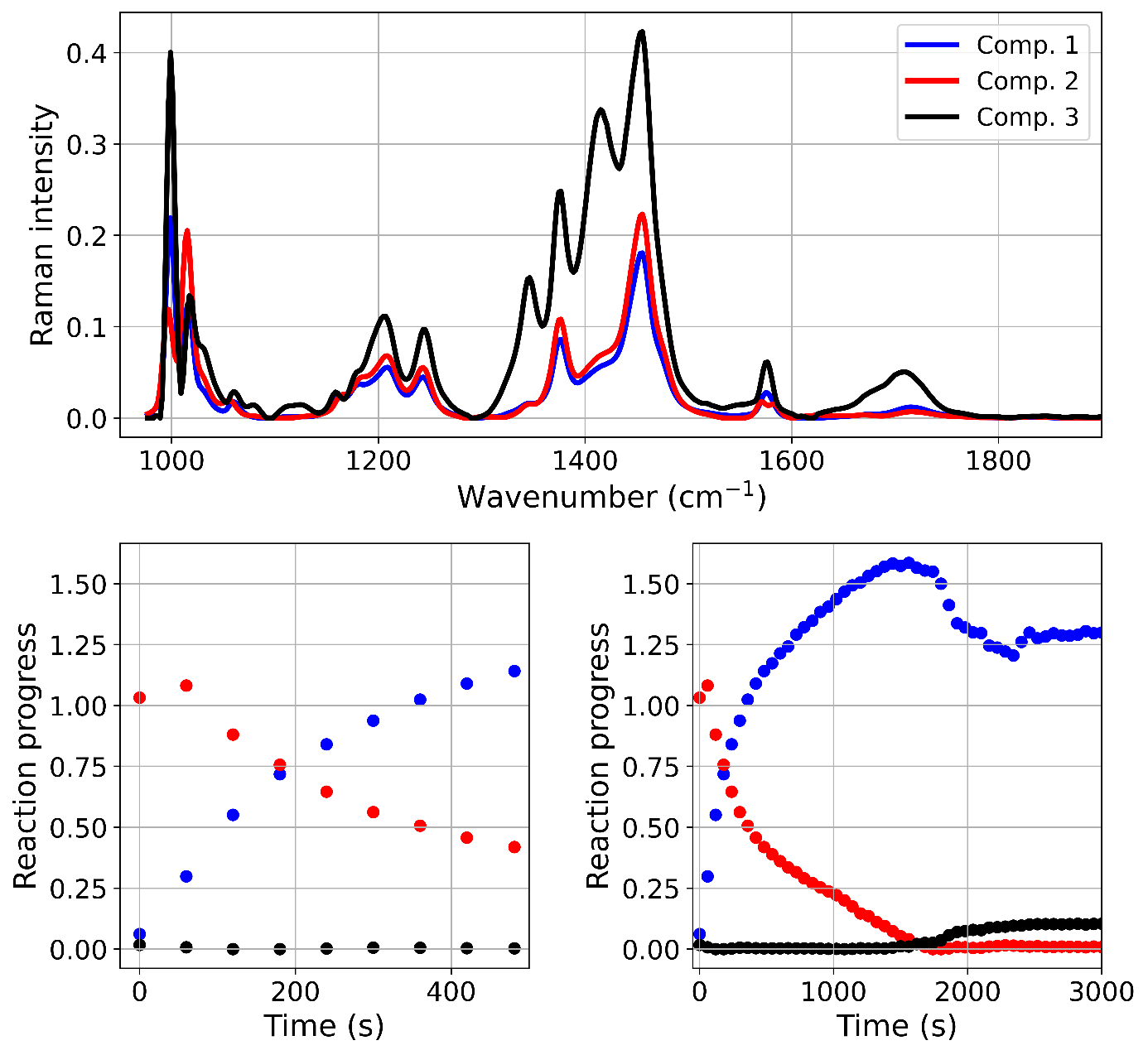


Figure S11. MCR analysis with three components.


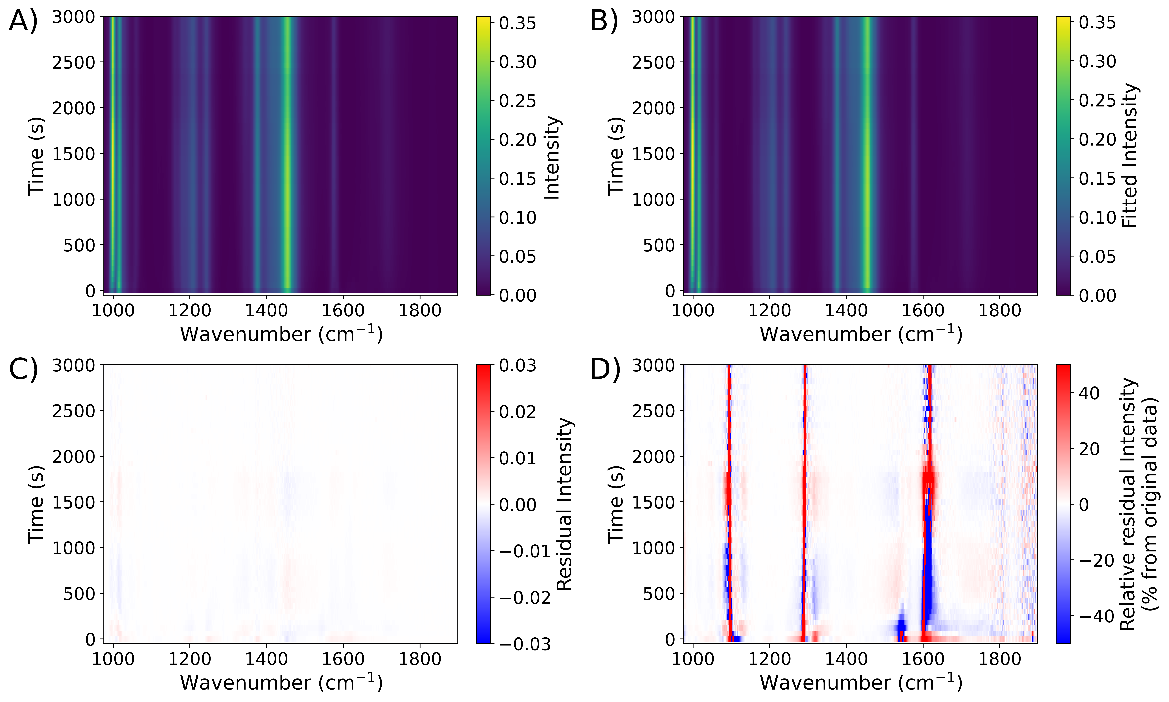


Figure S12. Error analysis with three components.


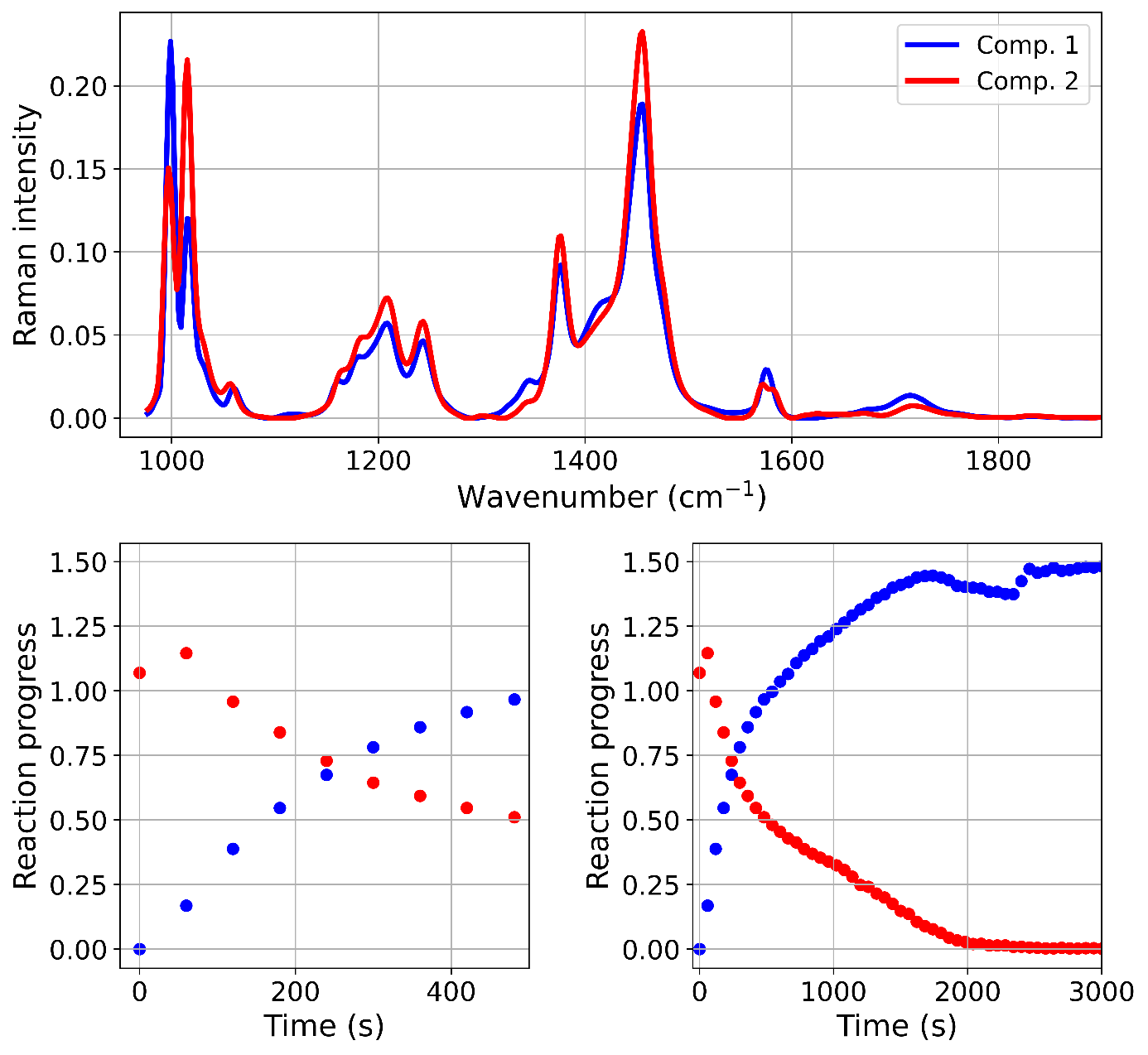


Figure S13. MCR analysis two components.


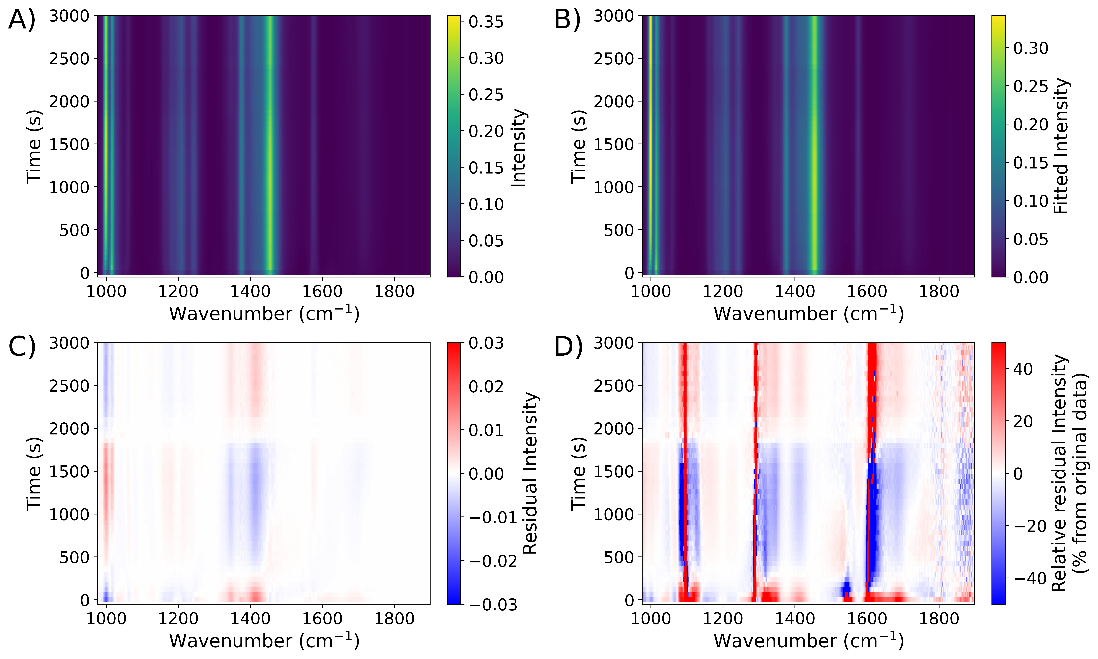


Figure S14. Error analysis with two components.

# 9. ^1^H-NMR Spectra

## 9.1 Ammoxidation

Figure S15. 1H-NMR (400 MHz, CD_3_OD) of nitrile **2.**

Figure S16. 1H-NMR (400 MHz, CD_3_OD) of nitrile **4**

Figure S17. 1H-NMR (400 MHz, CD_3_OD) of nitrile **5**

Figure S18. 1H-NMR (400 MHz, CD_3_OD) of nitrile **6**

Figure S19. 1H-NMR (400 MHz, CD_3_OD) of nitrile **7**

 Figure S20. 1H-NMR (400 MHz, CD_3_OD) of nitrile **8**

Figure S21. 1H-NMR (400 MHz, CD_3_OD) of nitrile **9**

 Figure S22. 1H-NMR (400 MHz, CD_3_OD) of nitrile **10**

Figure S23. 1H-NMR (400 MHz, CD_3_OD) of nitrile **11**

Figure S24. 1H-NMR (400 MHz, CD_3_OD) of nitrile **12**

 Figure S25. 1H-NMR (400 MHz, CD_3_OD) of nitrile **13**

 Figure S26. 1H-NMR (400 MHz, CD_3_OD) of nitrile **17**

 Figure S27. 1H-NMR (400 MHz, CD_3_OD) of nitrile **19**

## 9.2 Derivatizations

## 9.2.1 Functionalizations

 Figure S28. 1H-NMR (400 MHz, CDCl_3_) of tetrazine **22** Figure S29. 1H-NMR (400 MHz, CD_3_OD) of benzothiazole **23**

 Figure S30. 1H-NMR (400 MHz, CD_3_OD) of amidoxime **24**

## 9.2.2 Bioorthogonal Ligations

 Figure S31. 1H-NMR (400 MHz, D_2_O) of thiazoline **25**

The formation of the labile oxadiazoborole adduct **26** was followed *in-situ* by ^1^H-NMR (Figure S32). After the addition of one equivalent of phenylboronic acid, an equilibrium forms and unreacted **24** remains. The addition of a second equivalent of phenylboronic acid results in an equilibrium in which the starting material is mostly consumed (Figure S33).

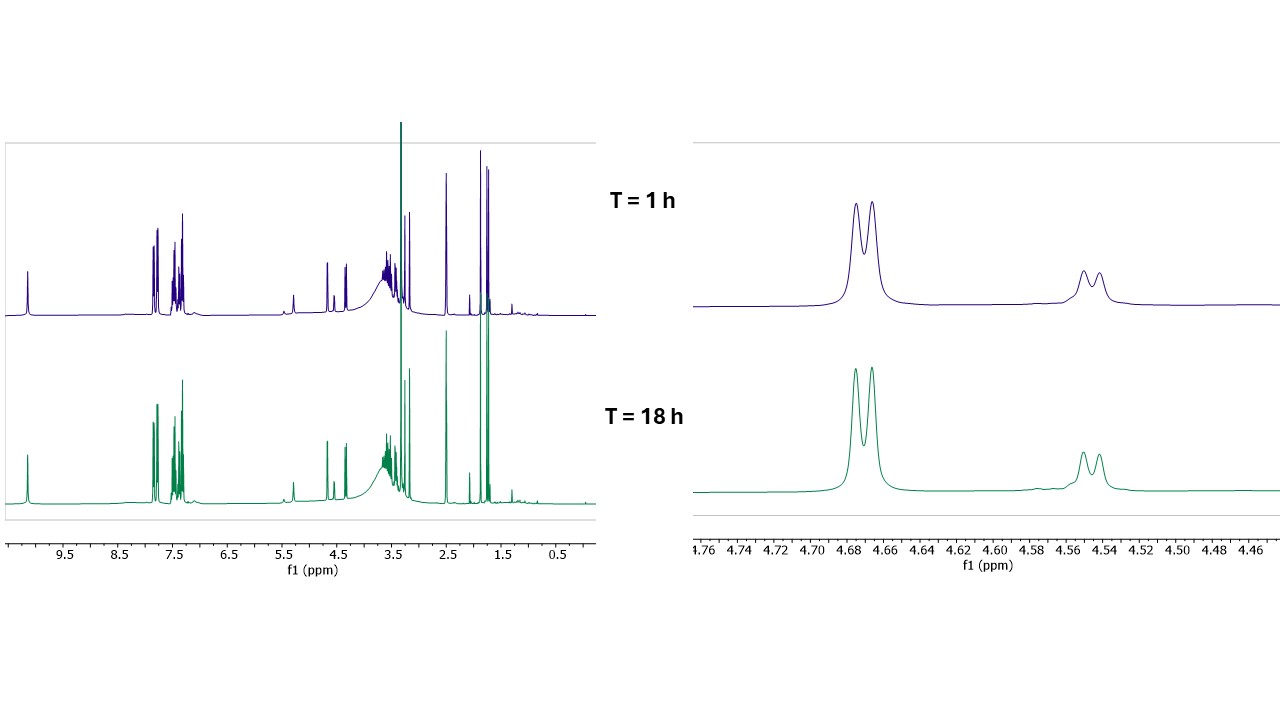


Figure S32 The formation of oxadiazoborole **26** followed over time by ^1^H-NMR (left) with a zoom in of the anomeric region (right).


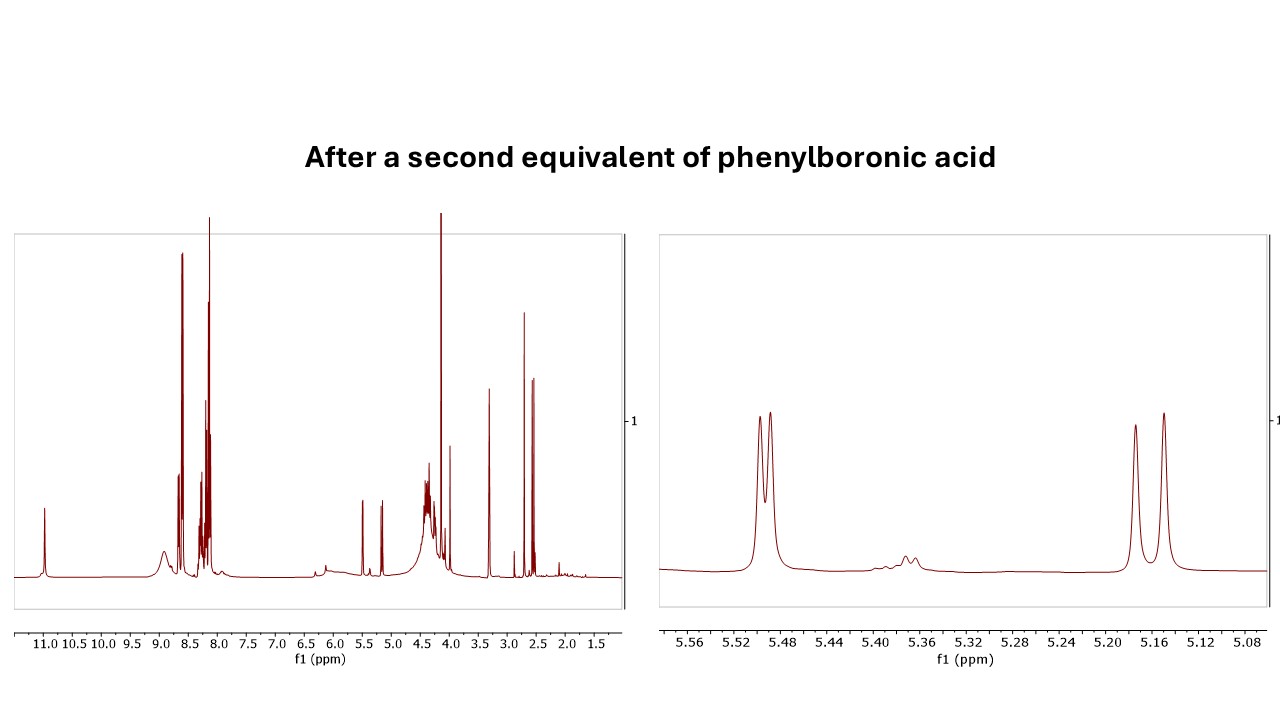


Figure S33 ^1^H-NMR spectrum of the reaction mixture of the formation of **26** after the addition of a second equivalent of phenylboronic acid (left) with a zoom in of the anomeric region (right).

# 10. ^13^C-NMR Spectra

## 10.1 Ammoxidation

 Figure S34. ^13^C-NMR (101 MHz, CD_3_OD) of nitrile **2.**

 *Figure S35. ^13^C-NMR (101 MHz, CD_3_OD) of nitrile* ***4.***

*Figure S36. ^13^C-NMR (101 MHz, CD_3_OD) of nitrile* ***5.***

 *Figure S37. ^13^C-NMR (101 MHz, CD_3_OD) of nitrile* ***6.***

 *Figure S38. ^13^C-NMR (101 MHz, CD_3_OD) of nitrile* ***7.***

 *Figure S39. ^13^C-NMR (101 MHz, CD_3_OD) of nitrile* ***8.***

 *Figure S40. ^13^C-NMR (101 MHz, CD_3_OD) of nitrile* ***9.***

*Figure S41. ^13^C-NMR (101 MHz, CD_3_OD) of nitrile* ***10.***


 *Figure S42. ^13^C-NMR (101 MHz, CD_3_OD) of nitrile* ***11.***

 *Figure S43. ^13^C-NMR (101 MHz, CD_3_OD) of nitrile* ***12.***

 *Figure S44. ^13^C-NMR (101 MHz, CD_3_OD) of nitrile* ***13.***

 *Figure S45. ^13^C-NMR (101 MHz, CD_3_OD) of nitrile* ***17.***

 *Figure S46. ^13^C-NMR (101 MHz, CD_3_OD) of nitrile* ***19.***

## 10.2 Derivatizations

## 10.2.1 Functionalizations

*Figure S47. ^13^C-NMR (101 MHz, CDCl_3_) of tetrazine* ***22.***

*Figure S48. ^13^C-NMR (101 MHz, CDCl_3_) of benzothiazole* ***23.***

 *Figure S49. ^13^C-NMR (101 MHz, CD_3_OD) of amidoxime* ***24.***

## 10.2.2. Bioorthogonal Ligations

 *Figure S50. ^13^C-NMR (101 MHz, D_2_O) of thiazoline* ***25.***

*Figure S51. ^13^C-NMR (101 MHz, DMSO-d6) of oxadiazoborole* ***26.***
